# Effect of Genetic Ancestry on Ecologically Important Fitness Traits in Hybridizing *Populus* Species: Relevance for Conservation and Forest Management

**DOI:** 10.1111/eva.70215

**Published:** 2026-03-25

**Authors:** Erik Szamosvári, Roman Ufimov, Nicola G. Criscuolo, Luisa Bresadola, Stefano Castiglione, Angela Cicatelli, Christian Lexer, Marcela van Loo

**Affiliations:** ^1^ Department of Forest Growth, Silviculture & Genetics Austrian Research Centre for Forests (BFW) Vienna Austria; ^2^ Department of Botany and Biodiversity Research, Faculty of Life Sciences University of Vienna Vienna Austria; ^3^ Department of Chemistry and Biology “A. Zambelli” University of Salerno Fisciano Italy; ^4^ Department of Biology University of Fribourg Fribourg Switzerland

**Keywords:** clonality, genetic ancestry, growth, hairiness, poplar, survival

## Abstract

Forest trees and their hybrids exhibit diverse adaptive strategies, reflected in functional traits that enhance fitness under varying biotic and abiotic stresses. Hybrid zones serve as natural experiments for studying evolutionary processes and have also garnered attention for their relevance in adaptive nature conservation and forest management, particularly in response to climate change and habitat fragmentation. However, the influence of genetic ancestry on ecologically relevant traits in hybrid zones remains insufficiently studied and understood. We investigated in this study the effects of genetic ancestry on juvenile survival, clonality, vegetative growth, and leaf hairiness in hybridizing *Populus alba* (L.), *Populus tremula* (L.), and their natural hybrid *Populus × canescens* (Aiton) Sm. Using genetic ancestry estimates derived from restriction site‐associated DNA sequencing and genotyping‐by‐sequencing, we analyzed plant material established from either seeds collected in different years or vegetative cuttings, grown across three common garden environments. We observed consistent patterns of seed sapling survivorship in two common gardens: backcrosses to *Populus tremula* and recombinant hybrid genotypes exhibited higher probability of juvenile mortality after the first 3 years compared to F_1_ hybrids, backcrosses to *P. alba* , and the parental species. Genetic ancestry also influenced clonal reproduction and early vegetative growth, with hybrids genetically closer to *P. alba* exhibiting enhanced growth and higher clonal rooting success. Plant propagation material from older seed collections tended to grow less and exhibited lower clonality. Additionally, leaf reflectance, a proxy for leaf hairiness, varied along the admixture gradient, with increasing *P. alba* ancestry corresponding to denser pubescence. Finally, we discussed the implications of these findings, particularly regarding pubescence, for forest breeding and restoration initiatives.

## 1 Introduction

Forest trees, due to their sessile nature and long lifespan, have developed a range of adaptation strategies to optimize fitness, tolerate biotic and abiotic stresses, and thrive in specific environments (Laughlin 2024). Such adaptations are manifested in functional traits, defined as morphological, physiological or phenological features measurable at the individual level (Violle et al. 2007). These also include rapid shoot and root growth, leaf pubescence and the ability to reproduce asexually via vegetative reproduction. Understanding these traits is pivotal not only for predicting ecosystem dynamics and tree responses to future climates with more extreme events, but also for guiding conservation efforts and forest management strategies, particularly those related to drought adaptation, pest management (Sharan et al. 2024), and breeding for climate‐resilient forests (O'Brien et al. 2017). Selecting fast‐growing genotypes with high survival rates promotes carbon sequestration, thereby contributing to climate change mitigation (Bastin et al. 2019). Furthermore, vegetative reproduction through shoot and root units or fragments, that remain attached to or are dispersed from the parent tree, supports population persistence and range expansion, with significant implications for genetic conservation and resilience in fragmented habitats (Lander et al. 2019; Lowry et al. 2024; Zhang et al. 2016). Additionally, leaf pubescence improves herbivore resistance, reduces water loss, regulates thermal balance, and minimizes UV radiation damage (Moles et al. 2020; Plett et al. 2010). Importantly, these adaptation strategies and traits not only mediate interactions between pure species and the environment, but also between the hybrids and their ecological settings.

Rapid advancements in genetics and genomics provide powerful tools for distinguishing hybrids from pure species and for characterizing the genetic admixture (ancestry) of the hybrids. These tools allow for precise estimation of allele proportions from ancestral populations or classification of hybrids into categories such as F_1_ hybrids, F_2_–F_n_ recombinants, and backcrosses to particular parental species (Bresadola et al. 2019; Christe et al. 2016). Consequently, studies investigating the effect of genetic ancestry on functional traits and their association can now become more detailed and insightful.

The importance of studying not only pure species, but also their hybrids lies in the fact that tree species frequently hybridize, or even form natural hybrid zones, which are well‐documented across economically and ecologically important genera such as *Salix* spp. (Pittet et al. 2025), *Quercus* spp. (Lazic et al. 2021), *Pinus* spp. (Menon et al. 2020), *Rhizophora* spp. (Francisco et al. 2018), *Eucalyptus* spp. (Pfeilsticker et al. 2022), and *Populus* spp. (Bolte et al. 2024). These hybrid zones are a cornerstone of evolutionary biology, providing natural experiments to study processes such as barriers to gene flow, adaptation and speciation (Harrison 1990; Hewitt 1988). Hybridisation and introgression are widespread across plants and can also generate ecologically consequential variation well beyond forest trees, as illustrated by genomic studies in sunflowers (*Helianthus*; Owens et al. 2021), monkeyflowers (*Mimulus*; Farnitano et al. 2025), alongside such herbaceous systems as Oxford ragwort (*Senecio*; Nevado et al. 2024) and *Arabidopsis* contact zones where introgression contributes to adaptation (e.g., Marburger et al. 2019; Scott et al. 2025). In the context of climate change and habitat fragmentation, understanding hybrid dynamics has gained increasing importance for adaptive conservation and forest management (Janes and Hamilton 2017; Lu et al. 2024; Reed‐Métayer et al. 2025). This understanding is particularly relevant when protecting species complexes rather than focusing solely on pure species, and for promoting admixture with more drought‐tolerant taxa to mitigate drought sensitivity (Zimmermann et al. 2025). Because selection on hybrid genotypes can vary across environments and through time, robust inference about hybrid survival and fitness benefits from replication across localities and temporal cohorts, ideally via independent field experiments (Gompert et al. 2017; Martin and Gould 2020; Zhang et al. 2020; Tataru et al. 2023).

The diploid genus *Populus* (Salicaceae) provides a tractable system for such replicated tests. In particular, the ecologically distinct *Populus alba* L. and *P. tremula* L. (white poplar and aspen, respectively) form a well‐characterised hybrid complex that combines extensive natural hybridisation with replicated field plantings across contrasting environments (Christe et al. 2016; Lexer et al. 2010; Lindtke et al. 2012). *P. alba* , a native tree growing almost exclusively in riparian areas of Eurasia and northern Africa, endures severe abiotic stressors including flooding, drought, and salinity (Dickmann and Kuzovkina 2014). In contrast, *P. tremula* , native to upland areas of temperate and boreal forests primarily in northern Eurasia, is frequently colonizing forest edges, open woodlands, and to a lesser extent riparian zones (Dickmann and Kuzovkina 2014). Despite their ecological divergence, manifested in differences in phytochemical, morphological (notably leaf hairiness) and growth traits (Caseys et al. 2015; Lexer et al. 2005), reproductive isolation between them is weak (Christe et al. 2016), facilitating hybridization where their ranges overlap, resulting in a continuum of *Populus* × *canescens* (Aiton) Sm. hybrid genotypes in seedlings (Lindtke et al. 2014). However, a study from Christe et al. (2016) on the survivorship of 4‐year‐old saplings in a common garden environment revealed a selective disadvantage for recombinant hybrids (F_n_), leading to a higher proportion of F_1_ hybrids, a pattern which the authors also found in natural hybrid zones. While some hybrids exhibit intermediate traits, others display transgressive variation, increasing the complexity of hybrid zones (Lindtke et al. 2012). Both parental species and their hybrids also reproduce vegetatively (asexually) in natural environments, predominantly through root suckers (Eckenwalder 1996), with *P. × canescens* even producing more ramets and occupying larger areas than *P. alba* within a natural hybrid zone in Austria (van Loo et al. 2008). For artificial vegetative reproduction in poplar breeding, however, shoot cuttings are predominantly used as starting material (Zhao et al. 2014).

In this study, we examined the effect of genetic ancestry on juvenile survival, the success of vegetative reproduction (clonality), vegetative growth and level of pubescence. We utilized previously obtained genetic ancestry estimates to characterize hybrid classes and parental species across three common gardens (CGs) located in different countries. Two of these CGs, established in earlier studies, provided survivorship data for 3‐year‐old saplings, allowing comparisons across two distinct environments. In the third CG, established in this study, we used vegetative material derived from seeds collected and germinated over three different years, enabling us to investigate how genetic ancestry influences clonality, vegetative growth, and pubescence. We specifically addressed the following questions:

- Did juvenile survival vary across common gardens for individuals with different ancestry?
- Did the year of seed germination influence clonality and growth patterns, and was there a relationship between genetic ancestry and these traits?
- Did leaf reflectance, as a proxy for hairiness, differ among hybrid classes, and was this variation consistent with genetic ancestry, particularly for hybrids related to *P. alba* ?

The results were then discussed in the broader context of their implications for conservation and forest management.

## 2 Material and Methods

### 2.1 Plant Material and Common Gardens

**FIGURE 1:**
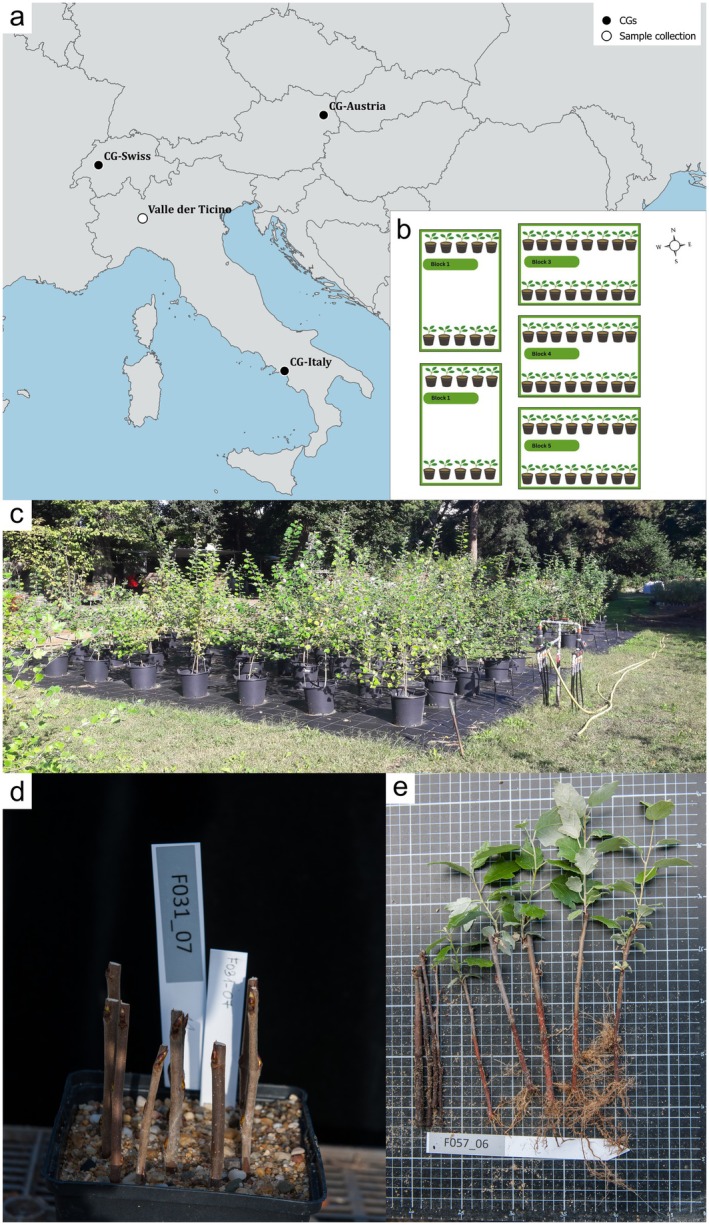
Overview of study sites and experimental design. (a) Geographic distribution of the hybrid zone used for seed sampling, and the three CGs with their locations in Fribourg (Switzerland), Salerno (Italy), and Vienna (Austria). (b) Schematic design of the common garden (CG‐Austria) at the Botanical Garden of the University of Vienna, illustrating the experimental layout with planting blocks. (c) Photo of CG‐Austria displaying the experimental setup and growing saplings in blocks 4 and 5 in September 2017. (d) Photo of cuttings from *P. tremula* taken in April 2016. (e) Photographic documentation for the clonality experiment for determining mortality and diameter covariate taken in July 2016.

We studied plant material from three common gardens (CGs); in Fribourg, Switzerland (CG‐Swiss, 46 47′ 30.6″ N 7°09′ 30.9″ E), Salerno, Italy (CG‐Italy, 40 46′ 55.9″ N 14 44′ 34.2 E), and Vienna, Austria (CG‐Austria, 48 11′ 37.7″ N 16 22′ 58.6″ E) (Figure 1a). These CGs included progeny from open‐pollinated maternal trees from a natural hybrid zone in the Parco Lombardo della Valle del Ticino on the Swiss‐Italian border, where both poplar parental species and their hybrids coexist (Lindtke et al. 2014).

Two CGs—CG‐Swiss, located at the Botanical Garden of the University of Fribourg (Switzerland) and CG‐Italy, located at the University of Salerno (Italy)—were established using seed collections from 2010 to 2011, and in the case of CG‐Swiss, also from 2014 (Bresadola et al. 2019; Lindtke et al. 2014). In total, approximately 500 seedlings from 39 families were grown in pots (Bresadola et al. 2019; Caseys et al. 2015). The seeds were germinated in the same year they were collected. Material from these CGs was used for studying mortality (survival) and clonality in our study.

In the spring of 2017, a third CG (CG‐Austria) was established at the Botanical Garden of the University of Vienna (Figure 1b) using cuttings of selected genotypes from CG‐Swiss for studying growth and leaf hairiness (leaf reflectance). The CG‐Austria consisted of 200 different genotypes (10 *P. tremula* , 20. *P. alba* and 170 of *P. × canescens* ) from 15 families/mother trees. These selected genotypes were propagated via root cuttings (185 genotypes) and shoot cuttings (15 genotypes). The cuttings were potted into 12‐l pots with a humus‐enriched substrate and arranged in a randomized block design with five blocks, each consisting of 40 genotypes: two *P. tremula* , 34 *P. × canescens* and four *P. alba* . Within each block, potted cuttings were spaced 1 m apart, with 2‐m spacing between blocks. An automated irrigation system was installed in early 2017. Apart from regular watering, the saplings were grown without additional interference (Figure 1c).

### 2.2 Fitness and Phenotypic Trait Measurements

#### 2.2.1 Survival of Seed Saplings

The survival of seed saplings germinated in 2010 and 2011 was recorded after 3 years of growth in two climatically different CGs (Figure S2): CG‐Swiss (*n* = 137) and CG‐Italy (*n* = 118) as binary data (alive = 1, dead = 0).

#### 2.2.2 Clonality

In early spring 2016, five or more shoot cuttings from 231 genotypes, originating from CG‐Swiss individuals, were cut into 15–20 cm long pieces, placed into labelled pots with a sandy soil mixture (Figure 1d), and grown in open‐air conditions at the Botanical Garden of the University of Vienna, Austria. At the beginning of July 2016, the number of surviving cuttings—those that remained green and fresh, and/or sprouted and/or produced roots—was recorded using photographic documentation (Figure 1e). In addition, photographs were first taken by placing the cuttings on a metric measuring and scoring board to account for variation in the initial cutting quality determined by cutting diameter (diameter covariate). The photographs were then visually assessed, classifying the starting material into five categories, ranging from 1 (very thin, < 3.5 mm) to 5 (very broad, > 6.5 mm), with intermediate categories scored linearly. A subset of the starting material was measured using the software Image J (https://imagej.nih.gov/ij/) to validate the accuracy of our scoring. Clonality (Equation 1) was quantified as “cutting success” and determined as the proportion of surviving cuttings per individual, which is the standard operational approach for producing planting stock:

(1) $$\text{Clonality}=\frac{\text{number of cuttings alive}}{\text{total number of cuttings}\;\mathrm{per}\;\text{genotype}}$$

We therefore interpret cutting survival as a proxy for clonal propagation capacity under management‐relevant conditions, rather than as a direct measure of natural clonal establishment via root suckering or vegetative spread in situ.

#### 2.2.3 Juvenile Vegetative Growth

Only plants derived from root cuttings (root‐cutting saplings) in CG‐Austria were included in this assessment. From April to October 2018, six measurements of stem diameter and tree height were recorded in 5‐week intervals. Stem diameter was measured with a digital caliper between the north‐ and south‐facing sides of the trunk, approximately 8 cm above the soil surface in the pots. Tree height was measured as the distance from the level of the soil in the pots to the top of the terminal shoot. As the terminal shoot of four individuals was damaged by wind, the tree height data were available for 181 trees in the final analysis. Relative growth rate (RGR) was calculated for each measured tree following the common Equation (2) of Fisher (1921):

(2) $$\mathrm{RGR}=\frac{\left(\log_{e}\left({\text{measurement}}_{2}\right)-\log_{e}\left({\text{measurement}}_{1}\right)\right)}{{\text{time}}_{2}-{\text{time}}_{1}}$$

Here, time_1_ and time_2_ refer to specific time points at which the measurements were taken, while measurement_1_ and measurement_2_ correspond to the recorded values for stem diameter or tree height at those time points. This RGR equation was chosen as it facilitates more meaningful comparisons than the absolute growth rate (Hunt 1990).

#### 2.2.4 Leaf Reflectance

At the beginning of July 2017, three fully expanded leaves, without any signs of herbivory, were collected from the terminal shoot of 198 trees in CG‐Austria, leaving out two trees that did not have enough undamaged leaves. The leaf samples were immediately labelled, pressed in a standard herbarium press, and dried for 14 days in an herbarium drying cabinet at 32°C with continuous air circulation. Dried leaves were digitized using a Canon 9000F Mark II scanner and the images were saved in TIFF format. Image exposure was standardized by manually adjusting black and white points of each sample using a Kodak CAT 1527654 color standard, following Lexer et al. (2009). To minimize bias, abaxial leaf surfaces were scanned in a random order using consistent settings: automatic sharpness, color mode, 300 dpi resolution, and a black background. The scanned 8‐bit images were then analyzed in open‐source software Image J. Mean, modal, and median grey values of a designated circular area of each leaf were measured between primary and secondary veins adjacent to the main vein, providing an estimate of leaf hairiness (Lexer et al. 2009) and, consequently, trichome density (Plett et al. 2010). The mean grey values of leaves, representing the average pixel intensity within the selected area: that is, the sum of all pixel values divided by the number of pixels, originating from the same individual were then merged to an average value per tree, which was employed for further statistical analyses.

### 2.3 Genetic Background of Plant Material

During this study, we relied on previously estimated genotyping resources for the individuals used here. Specifically, we had access to two reduced‐representation SNP datasets generated with different library preparations. The first dataset was produced using a GBS protocol based on *EcoR*I and *Mse*I digestion (Lindtke et al. 2014; following Parchman et al. 2012) and is referred to here, as in the original publication, as the “GBS” dataset. Libraries were sequenced single‐end on an Illumina HiSeq 2000. After read curation and barcode processing, reads were aligned to the *P. tremula* draft genome assembly v0001 (UPSC draft genome release, http://popgenie.org; http://loblolly.ucdavis.edu/bipod/ftp/Genome Data/genome/Pota/) using BWA (Li 2013), and variants were called with SAMtools/BCFtools (Danecek et al. 2021), yielding genotype likelihoods for biallelic sites. SNPs were retained after filtering for site probability, coverage across individuals, minor allele frequency (< 0.05), and related quality criteria, resulting in 11,976 SNPs used for downstream ancestry inference.

The second dataset was generated using a PstI‐based RAD‐seq protocol (Bresadola et al. 2019), following standard RAD‐seq approaches (e.g., Baird et al. 2008), and is referred to here as the “RAD‐seq” dataset. RAD libraries were sequenced single‐end on Illumina HiSeq 2500. Reads were mapped to the *P. trichocarpa* reference genome (Ptrichocarpa_210_v3.0; Tuskan et al. 2006) using Bowtie2 (Langmead and Salzberg 2012), variants were called with GATK (DePristo et al. 2011), and sites were filtered to retain reliable biallelic SNPs (e.g., removing indels and sites near indels), excessive‐depth loci indicative of paralogy, and sites with high missingness and/or low minor allele frequency (< 0.05). After filtering, 127,322 SNPs were retained for genome‐wide ancestry inference.

For both datasets, two ancestry metrics estimated in the original studies were used here without modification: the genome‐wide admixture proportion *q* (proportion of *P. alba* ancestry, with the complementary fraction deriving from *P. tremula* ) and the interspecific ancestry parameter *Q*_*12*_ (proportion of the genome carrying heterospecific ancestry, informative about hybrid generation).

Genome‐wide ancestry and *Q*_*12*_ from the RAD‐seq data (Bresadola et al. 2019) were inferred using *entropy* (Gompert et al. 2017), which estimates ancestry directly from genotype likelihoods; Bresadola et al. (2020) additionally evaluated and corrected RAD‐seq–specific biases (notably under‐calling of heterozygotes) by recalibrating genotype likelihoods prior to ancestry estimation. In the GBS dataset, Lindtke et al. (2014) estimated *q* and *Q*_*12*_ using a custom Bayesian clustering framework conceptually similar to *entropy* but explicitly integrating genotype uncertainty from sequence data and leveraging sibship structure to improve inference in open‐pollinated families. Full descriptions of the genotyping protocols and analysis are provided in Lindtke et al. (2014) and Bresadola et al. (2019, 2020).

Although plants in the CG‐Austria were not genotyped independently in the cited studies, they represent clonal replicates of individuals from the CG‐Swiss and therefore share the same genotypes and ancestry estimates. For analyses of clonality, growth, and leaf reflectance, we prioritised ancestry estimates derived from the RAD‐seq dataset (Bresadola et al. 2019), whereas survival analyses were based on the GBS‐derived ancestry estimates (Lindtke et al. 2014). This choice for survival was deliberate: it enabled direct comparability with the earlier survival analysis of Christe et al. (2016) at the CG‐Swiss, which used the same GBS‐based ancestry estimates, a comparable age (4 years old), and similar statistical approach. Importantly, ancestry estimates derived from GBS and RAD‐seq were never pooled or merged within a single analysis; each trait‐specific analysis relied on one dataset only. A direct cross‐validation of the GBS‐ and RAD‐seq‐based ancestry estimates, including the effects of genotyping error and their consequences for *q* and *Q*_*12*_, is provided in Bresadola et al. (2020). Bresadola et al. (2020) showed that estimates of *q* are largely insensitive to typical genotyping error, whereas raw RAD‐seq genotype calls can underestimate heterozygosity‐related quantities affecting *Q*_*12*_. This issue, however, is mitigated when genotype likelihoods are recalibrated, which was applied for the values used in the present study. Therefore, we believe any potential bias is negligible in this context.

**FIGURE 2:**
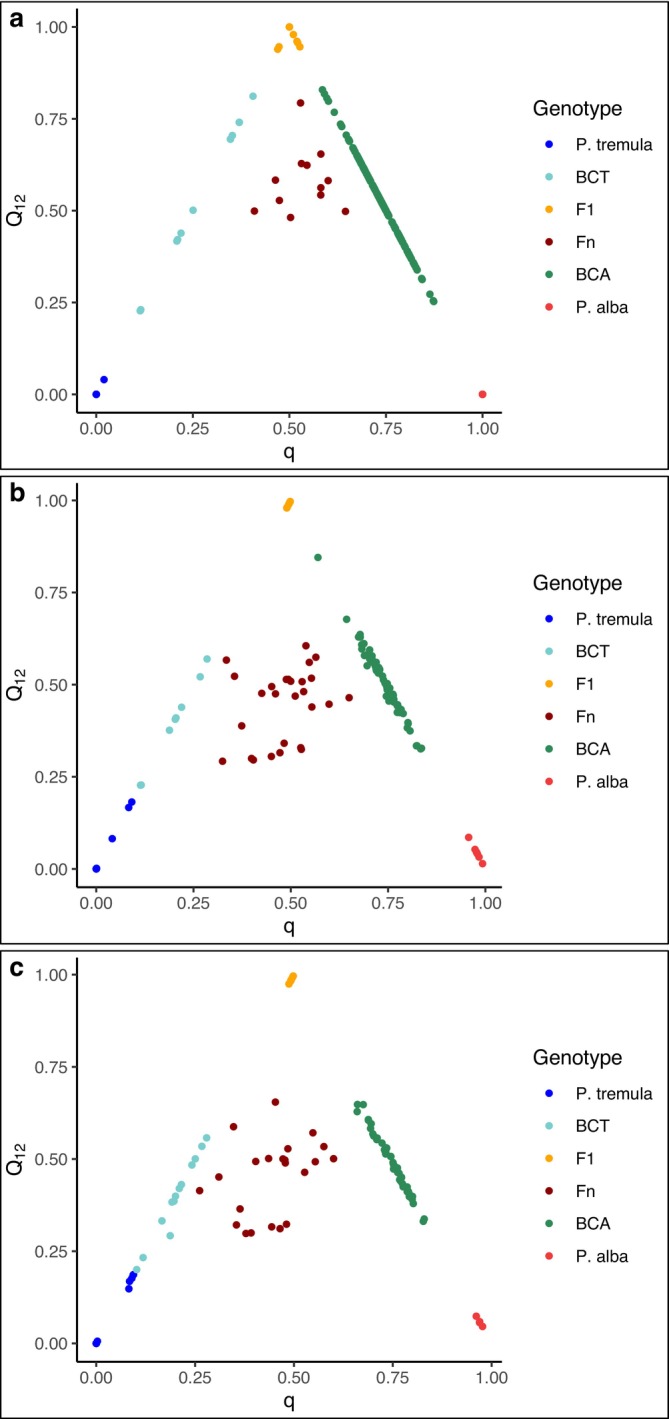
Plots of genetic ancestry parameters (*q* and *Q*_*12*_) of studied individuals in three common gardens (CGs). For (a) CG‐Austria, *q* and *Q*_*12*_ values were estimated using RAD‐seq data (Bresadola et al. 2019). For (b) CG‐Swiss and (c) CG‐Italy, values were derived from GBS data (Lindtke et al. 2014). BCA, backcrosses towards *P. alba* ; BCT, backcrosses towards *P. tremula* ; F_1_, first generation hybrids; Fn, recombinant hybrids as individuals belonging to subsequent generations.

For descriptive purposes (Figure 2), we interpreted *q* as follows: individuals with *q* ≤ 0.1 were assigned to *P. tremula* , those with *q* ≥ 0.9 to *P. alba* , and individuals with intermediate values (0.1 < *q* < 0.9) were classified as hybrids (*P. × canescens*) (Lexer et al. 2005; Lindtke et al. 2012, 2014). The second ancestry parameter, *Q*_*12*_ (range 0–1), was used as an indicator of hybrid generation: values of 0.9–1.00 are consistent with first‐generation hybrids (F_1_), whereas lower values indicate recombinant generations and/or parental‐like backcrosses, reflecting the expected reduction in interspecific heterozygosity with successive recombination (Buerkle and Lexer 2008; Milne and Abbott 2008). To separate recombinants generation hybrids (F_n_) from backcross‐like individuals for visualisation, we compared observed interspecific ancestry (*Q*_*12*_) to the F_1_ expectation given *q*. Under a simple two‐source model, the expected interspecific ancestry for an F_1_ is approximately *2q* for *P. alba* –biased genotypes (*q* ≤ 0.5) and 2(1−*q*) for *P. tremula* –biased genotypes (*q* > 0.5); recombination in later generations reduces *Q*_*12*_ below this expectation. We therefore classified individuals as F_n_ when *Q*_*12*_ was at least 0.1 lower than the corresponding benchmark (i.e., *Q*_*12*_ ≤ 2*q* – 0.1 or *Q*_*12*_ ≤ 2(1 – *q*) – 0.1); otherwise, they were classified as backcrosses. Because hybrid individuals in this system form a genomic continuum rather than discrete categories (Lexer et al. 2005; Christe et al. 2016), these thresholds are used only for visualisation and contextual interpretation. All statistical analyses treated *q* and *Q*_*12*_ as continuous covariates, such that the reported patterns reflect continuous ancestry effects rather than artefacts of categorical grouping.

### 2.4 Statistical Analyses

Data visualization and statistical analyses of fitness traits including survival, clonality, growth, and hairiness in relation to genetic ancestry (*q* and *Q*_*12*_) were performed in the open‐source software environment R (R Development Core Team 2008). The significance threshold for *p*‐values was set at *α* = 0.05, and all model assumptions were verified prior to conducting and interpreting the analyses.

#### 2.4.1 Survivorship

Generalized linear mixed‐effects models (GLMMs) were implemented using the *lme4* package (Bates et al. 2014) to assess the relationship between survivorship and genetic ancestry in two common gardens. In these models, predictor variables included both the linear and quadratic terms for the hybrid index (*q*) and the inter‐source ancestry index (*Q*_*12*_), while maternal family—classified by mother trees (*n* = 15)—was treated as a random factor, as it indicated a better balance between goodness of fit and model complexity than germination years of propagation sources. A series of models were constructed sequentially using the forward stepwise selection method (Field et al. 2012), starting with a GLMM containing only the intercept and then progressively adding the *q* term, *Q*_*12*_, the squared term for *q* (*q* ^*2*^), and finally the squared term for *Q*_*12*_ (*Q*_*12*_^*2*^), and the CG locations. This approach was applied consistently across all following models. Model selection was performed using analysis of variance (ANOVA) and the Akaike Information Criterion (AIC) to identify the best‐fitting model. The significance of fixed effects was determined by evaluating the 95% confidence intervals of their coefficients, which helped interpret which predictor variable contributed most to predicting survivorship.

#### 2.4.2 Clonality

GLMMs were again employed using the *lme4* package to assess the relationship between clonability (measured as cutting success) and genetic parameters. In these models, the genetic ancestry parameters (*q* and *Q*_*12*_) and a scored diameter covariate were included as fixed predictor variables, while cutting success, calculated from binary data, served as the response variable. The same forward stepwise model selection approach was used as in the survivorship analysis, with model selection and evaluation of coefficient significance following the procedure described above. In addition, the predicted probability across the full range of *q* values was later calculated separately for each year, due to the uneven distribution of *P. tremula* samples across the germination years of propagation sources.

#### 2.4.3 Juvenile Growth

First, Spearman's rank correlation was conducted to determine the relationship between the relative growth rate (RGR) of trunk diameter and the RGR of root‐cutting sapling height. The same was done for absolute height. We proceeded with height only, as these variables were highly correlated. Linear mixed‐effects models (LMMs) were applied using the *nlme* R‐package (Pinheiro et al. 2018) to analyze the effect of genetic ancestry on growth. Four different modelling approaches were tested with three distinct response variables. (i) In the first approach, absolute height measurements taken monthly for all trees (with April as the baseline) were modelled to reflect growth over time. Fixed predictor variables in this model included both genetic parameters (*q* and *Q*_*12*_), the germination year of the propagation source (2010, 2011, and 2014), and linear, quadratic, and cubic values representing the measurement time points (*n* = 6). The common garden block (location in the CG) was incorporated as a random factor. (ii) In the second approach, the monthly RGR—calculated using the April height as a reference—was used as the response variable, with the same fixed predictors and random factor applied. (iii) In the third approach, the overall RGR for the growing season of 2018 was modelled; here the time‐point values were removed from the fixed predictors because only one period was under investigation. (iv) In the final approach, overall RGR data were analyzed after removing trees categorized as pure *P. alba* and *P. tremula* , thereby focusing solely on hybrid genotypes to explore the impact of genetic ancestry and recombination. For all these models, AIC values extracted from ANOVA were used to select the best‐fitting models, and the significance of the coefficients was assessed accordingly.

#### 2.4.4 Pubescence

Linear regression models (LMs) were used to explore the genetic influence on leaf reflectance as a proxy for trichome density. Leaf reflectance, represented by the average grey values measured across replicate leaves per tree, resulted in a dataset of 198 statistical units. The regression models included *Q*_*12*_ and both the linear and quadratic terms for *q* as predictor variables. Models were built using a forward stepwise selection approach, as before, starting with an LM that contained only the intercept, then sequentially adding the *q* term, the *q* ^*2*^ term, and finally the *Q*_*12*_ term. Model selection was based on the significant decrease in the Residual Sum of Squares (RSS) between models, as evaluated using the F‐statistics (with a *p*‐value threshold of < 0.05). Once a final model was established, the significance of the predictor coefficients and their 95% confidence intervals was estimated.

## 3 Results

### 3.1 Survival of Seed Saplings

**FIGURE 3:**
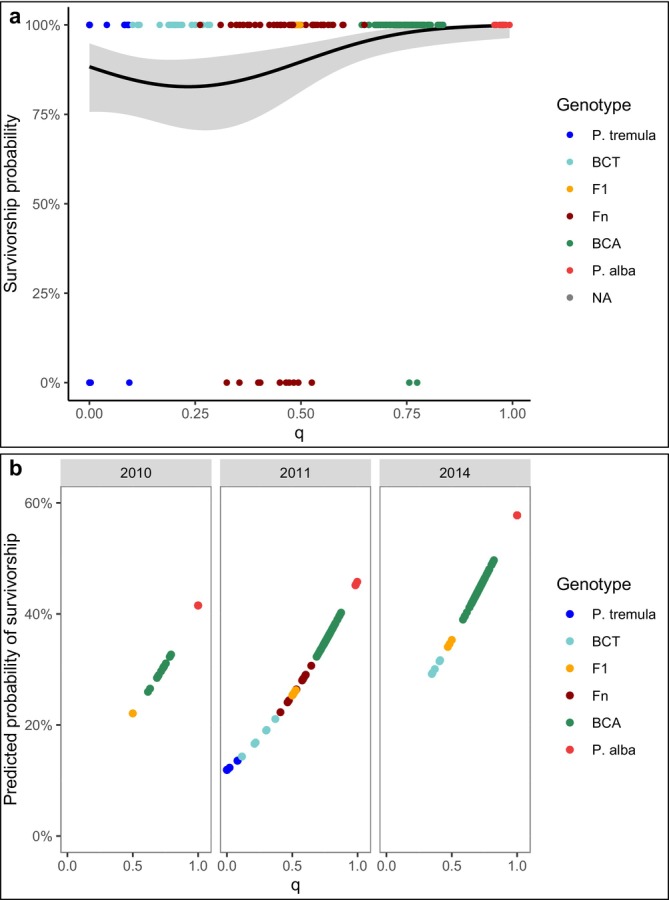
Effects of genetic ancestry on survival and clonal propagation. (a) Polynomial logistic curve with 95% CI illustrating the relationship between admixture index (*q*) and third‐year survivorship in two CG locations, reflecting the non‐linear effect of genetic ancestry on survival probability. (b) Predicted probabilities of clonal propagation success (probability of clonal cutting survivorship) as a function of admixture index (*q*), stratified by germination year of the propagation source (GY) illustrating the influence of genetic ancestry and GY source on the likelihood of successful clonal reproduction.

Genetic indices were assessed for their impact on the survival of poplar seed saplings using complementary ancestry estimates from a GBS study by Lindtke et al. (2014). The dataset included observations collected 3 years after planting at two distinct CG locations. The binary survival dataset (alive/dead) included 255 observations, consisting of 137 samples from CG‐Swiss and 118 from CG‐Italy. Of these, 236 seed saplings survived, while 19 seed saplings (8.05%) were recorded as dead over the 3 years. The best‐fitting GLMM, identified through ANOVA‐based model comparison (Table S1), included linear and quadratic values of hybrid index (*q*) and linear values of inter‐source ancestry (*Q*_*12*_) (ANOVA of GLMM: ΔAIC = 21.95, *p*‐value = < 0.0001). The evaluation of fixed coefficients of the best‐fitting model demonstrated that all predictor variables had a significant effect on seed sapling survivorship (Table S2). Incorporating the climatically different CG locations as a fixed predictor did not reduce the AIC value (Table S2), indicating no significant differences in proportion of survivorship between the CGs in Italy and Switzerland. This result holds despite the sites being situated in distinct Köppen–Geiger climate zones, with distinct patterns reflected in the monthly temperature and precipitation data (Figure S1). CG‐Italy falls within a Mediterranean climate (Csa), characterized by hot, dry summers and mild, wet winters, while CG‐Swiss lies in a temperate oceanic climate (Cfb), marked by moderate temperatures and evenly distributed precipitation throughout the year (Peel et al. 2007). The positive effect of *Q*_*12*_ indicates higher chances of survivorship with increased *Q*_*12*_ values, implying F_1_ hybrids had a greater likelihood to survive than subsequent recombinants (Figure S2). Additionally, the quadratic effect of *q* was positive, reflecting a lower survival probability for individuals with low to intermediate admixture index values, that is, backcrosses to *P. tremula* or as in our case later‐generation saplings (Figure 3a). These results suggest that recombinant hybrids with a genetic affinity to *P. tremula* suffered most from mortality.

### 3.2 Clonality

Here, we investigated the genetic factors influencing the success of shoot cuttings of individual genotypes to propagate asexually in a CG environment, using RAD sequencing data (Bresadola et al. 2019). Initially, ANCOVA was conducted to assess the role of the germination year of the propagation source (GY), which was subsequently treated as a random factor due to its significant interaction with both the admixture index (*q*) (Pr(<|z|) < 0.0001) and the inter‐source ancestry index (*Q*_*12*_) (Pr(<|z|) = 0.0031). Model selection based on AIC values identified the best‐fitting model when *q* was included as a fixed predictor and GY as a random factor (ANOVA, ΔAIC = 88.36, *p* < 0.0001; Table S3). Statistical analyses revealed that the admixture index (*q*) (Table S4) and the germination year of the propagation source (GY) significantly influence the propensity for asexual reproduction via shoot cuttings in a CG environment, with *P. tremula* being less successful when compared to *P. alba* (Figure 3b). Hybrids genetically closer to *P. alba* (with *q* approaching its maximum of 1.0) demonstrated increased cutting success, as evidenced by the higher survival of shoot cuttings. While variation in GY was evident in the dataset, *P. tremula* individuals, associated with lower cutting success, were unevenly distributed across sampling years. Consequently, their predicted probability was generalized across the entire range of *q* values (Figure S3). This generalization suggests that clonability is lowest at the minimum admixture index (*q* = 0.00) and when older propagation sources are used.

### 3.3 Juvenile Vegetative Growth

**FIGURE 4:**
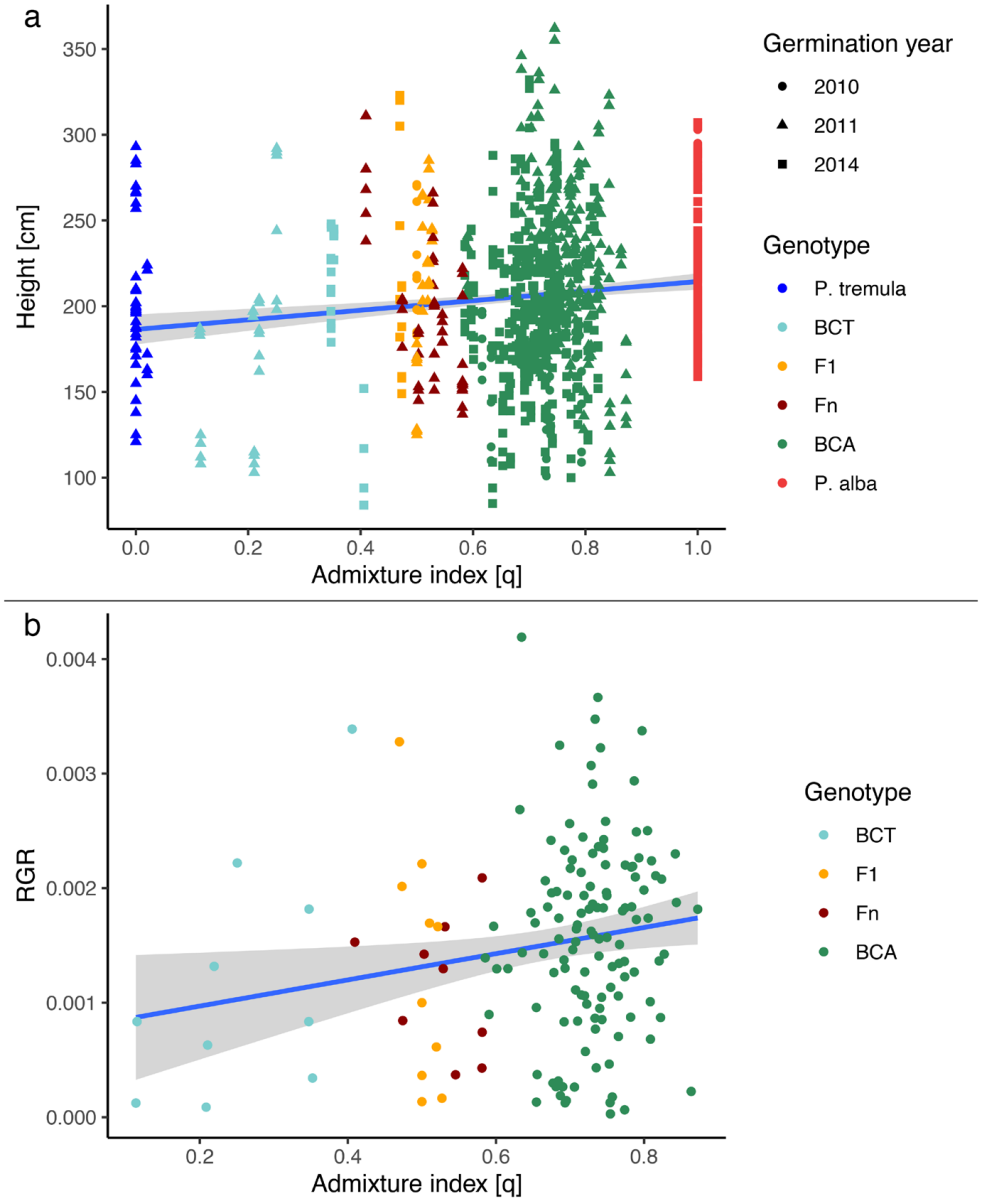
Influence of genetic ancestry on growth. (a) Linear regression line with 95% CI showing the relationship between all height measurements during the vegetation period and admixture index (*q*), illustrating the effect of genetic ancestry on all tree height measurements over the vegetation period. (b) Scatterplot with 95% CI for overall relative growth rate (RGR) in hybrids, highlighting the variability in RGR across hybrid individuals and the influence of genetic ancestry on growth.

Root‐cutting sapling height varied across genotypes during the 2018 vegetation period, ranging at the last measurement from 115 to 362 cm, with a mean height of 231.1 cm and a standard deviation of 46.51 cm. The best‐fitting LMM, identified through ANOVA, included GY, *q*, and linear, quadratic, and cubic terms of measurement time points as fixed predictor variables, with block position in the CG as a random factor (ANOVA of LMM, ΔAIC = 9.1, *p* value = 0.0009, Table S5). The positive effect of admixture index *q* (Table S6) indicated increased height of individuals genetically closer to *P. alba* (Figure 4a). Conversely, GY had a negative effect on height, indicating that root‐cutting saplings from older propagation sources tended to grow less (Table S6). Height changes over time followed a typical growth trajectory, with slower growth at the beginning and end of the vegetation period (Figure S4). Additionally, block position in the CG also influenced growth, as individuals in different blocks varied in height (Figure S5). In our second approach, we changed the response variable to RGR calculated for each month, using April as the reference point, while maintaining the same model structure as in the height analysis. Given the strong correlation of RGR calculated between height and diameter (Figure S6) (Spearman's rank test, *p*‐value < 2.2e‐16, *ρ* = 0.5898), subsequent RGR analyses were based on height measurements only. After comparison of LMMs, our best fitting model (ANOVA, ΔAIC = 15.96, *p*‐value < 0.0001, Table S7) only included linear, quadratic and cubic effects of time and block position in CG as a random factor. Neither genetic parameters nor GY caused a significant change when introduced in the models. In our third approach, RGR was calculated for the vegetation period of 2018 to see an overall picture of growth during this time. The best fitting model only included intercept and block number in CG as a random factor (ANOVA, ΔAIC = 26.07, *p*‐value < 0.0001, Table S8). None of the known genetic parameters brought significant changes in model comparisons. Graphical inspection (Figure S7) suggested merit in the removal of individuals of the pure parental species from the dataset to bring out patterns among hybrid individuals more clearly. Therefore, in the fourth and last approach, we ran another LMM comparison with the same model structure as previously, without the parental individuals in the dataset. The best fitting model included admixture index as fixed predictor variable and block position as a random factor (ANOVA, ΔAIC = 5.35, *p*‐value = 0.0067, Table S9). A positive effect of closer relatedness to *P. alba* on RGR was clearly demonstrated from the analysis of hybrid individuals (Figure 4b; Table S10). Further examination of RGR across blocks (Figure S8) indicated block‐specific differences, with Block 4 showing markedly increased RGR for BCAs, whereas differences were more moderate in other blocks.

### 3.4 Leaf Reflectance

**FIGURE 5:**
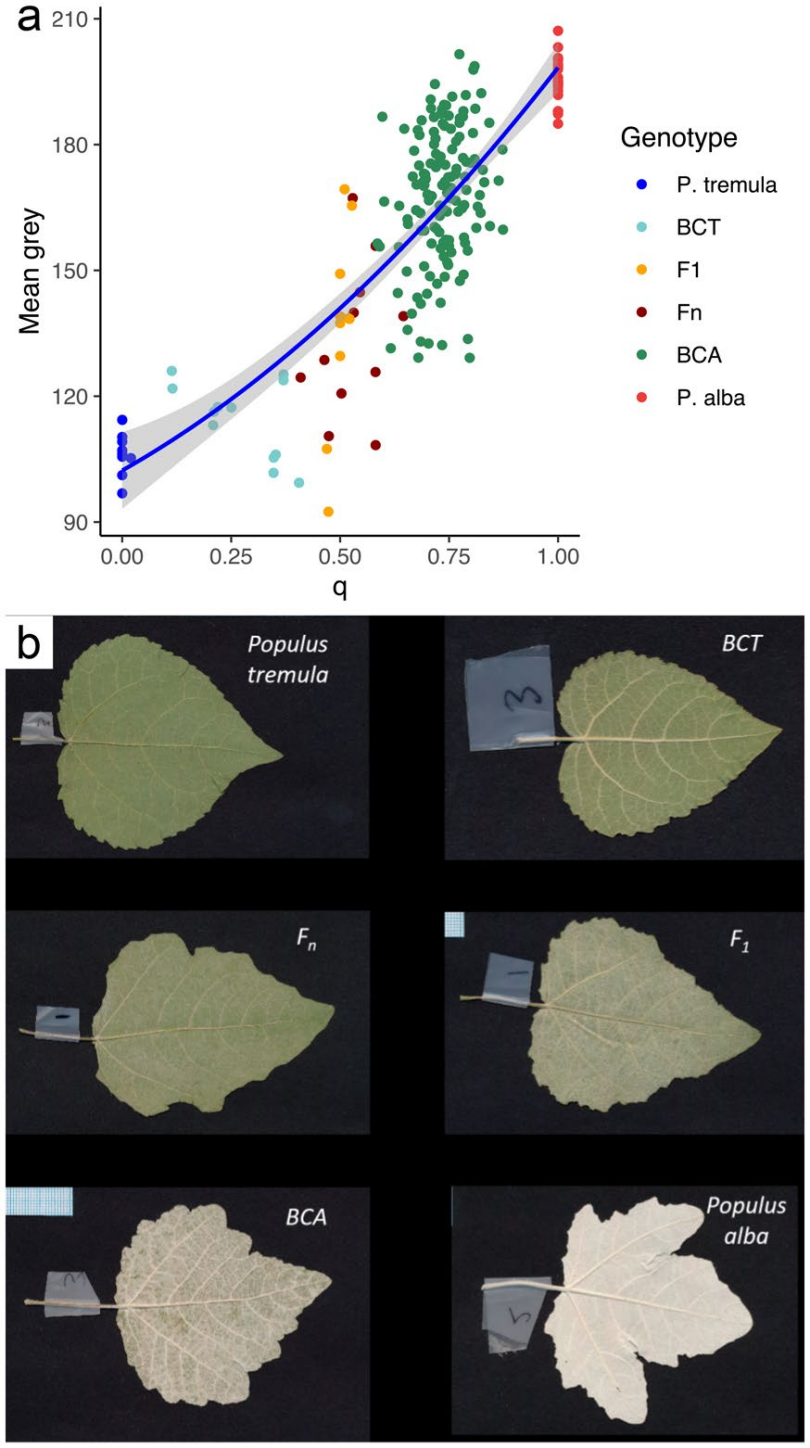
Relationship between genetic ancestry and leaf hairiness. (a) Polynomial regression curve with 95% CI illustrating the non‐linear relationship between admixture index (*q*) and mean grey values, reflecting the effect of genetic ancestry on hairiness. (b) Scanned leaf images of different genotypes exemplifying variation in abaxial leaf hairiness.

Our analysis of abaxial hairiness confirmed that genetic ancestry indices influence leaf reflectance. Measurements of mean, modal and median grey values for individual trees were highly correlated (Pearson correlation coefficient *r* > 0.99 in all variable pairs, Table S11). Given this strong correlation, mean grey values were used in all subsequent analyses. Model selection using ANOVA of mixed models indicated no significant effect of block arrangement on the response variable, as reflected by the non‐significant change in AIC values (ANOVA, ΔAIC = −1.79, *p*‐value = 0.6465, Table S12). Therefore, to identify the best‐fitting model, linear models were constructed and compared using ANOVA and F‐statistics (Table S13). The inclusion of *q* as a predictor decreased the residual sum of squares (RSS), and further introduction of the quadratic term of *q* resulted in an even lower RSS with a statistically significant effect (*p*‐value < 0.05, Table S14). Inclusion of *Q*_*12*_ did not result in significant changes during model comparison. Consequently, the best‐fitting model incorporated a polynomial function of admixture index (*y ~ x + x* ^*2*^) to explain variability in abaxial leaf hairiness. The significance of coefficients for fixed predictors of the best fitting model (Table S15) allows us to conclude that both linear and quadratic values of *q* had significant effects on the measured and merged mean grey values (Figure 5a). These positive effects of *q* indicate that BCA exhibit increased trichome density, as expected (Figure 5b).

## 4 Discussion

Hybridization plays a critical role in shaping genetic diversity, adaptation, and ecological interactions in forest tree species (Aitken et al. 2008; Barton 2001). Our findings highlight how genetic ancestry influences juvenile survivorship, clonality, growth, and leaf hairiness in *Populus* hybrids, providing new insights into postzygotic natural selection and hybrid persistence. We observed a consistent pattern across these traits, with hybrids genetically closer to *P. alba* exhibiting higher survival rates, greater clonal reproduction, and superior growth performance among hybrids, indicating a potential selective advantage of *P. alba* ancestry. In contrast, backcrosses to *P. tremula* and recombinant hybrids showed lower probability of survival, poorer clonal propagation, and reduced height, accompanied by the expected decrease in abaxial leaf hairiness. These results underscore the strong influence of genetic ancestry on early‐life fitness, offering a potential explanation for the genetic composition of natural hybrid populations. Finally, our study opens new avenues for incorporating hybrids into conservation and forest management strategies, which traditionally prioritize pure species (Allendorf et al. 2001; Jackiw et al. 2015).

### 4.1 Similar Patterns of Seed Sapling Survivorship Across Two Common Gardens

Despite the high seed production typical of *Populus* species, seedling mortality is substantial, and survival rates often vary among genotypes (Dickmann and Kuzovkina 2014; Lindtke et al. 2014; Scaracia‐Mugnozza et al. 1997; Schweitzer et al. 2002). Our analysis of juvenile survivorship in 3‐year‐old seed saplings across two climatically distinct common gardens (CG‐Italy and CG‐Swiss, Figure S1) underscores the pivotal role of genetic ancestry in determining survival outcomes. Higher probability of survival was observed in *P. alba* seed saplings, F_1_ hybrids, and backcrosses toward *P. alba* , while recombinant hybrids and backcrosses to *P. tremula* showed elevated mortality rates (Figure 3a). These patterns mirror the findings by Christe et al. (2016), who observed similar trends in 4‐year‐old saplings grown in CG‐Swiss. Their study likewise highlights the superior early‐life performance, in terms of survival, of *P. alba* ‐biased genotypes, with reduced fitness among more recombinant types, potentially due to disrupted gene interactions or genetic incompatibilities (Birchler 2003).

Juvenile selection in hybrid species is likely influenced by a complex interplay of genetic mechanisms that affect survival, stress tolerance, growth and adaptation, among others. The seedling stage is a critical developmental phase for any plant, due to high selection pressure resulting also from limiting factors and competition with other organisms (Ahmad et al. 2025). In addition to these challenges, recombinant genotypes frequently exhibit reduced viability due to negative epistatic interactions, where novel combinations of non‐coadaptive alleles from divergent parental species disrupt developmental or physiological processes (Phillips 2008). A specific form of such negative epistasis is described by the Bateson–Dobzhansky–Muller model, which explains how incompatible gene interactions can lead to postzygotic reproductive barriers and early‐life maladaptation in hybrids (Coyne and Orr 2004; Macaya‐Sanz et al. 2011). In later‐generation hybrids, recombination may unmask deleterious alleles, further reducing the fitness of hybrid progeny (Lindtke et al. 2014). More broadly, these postzygotic barriers can result from the disruption of coadapted gene complexes, where mismatched gene products fail to interact properly, resulting in dysfunctional phenotypes (Lindtke and Buerkle 2015). Asymmetric introgression is often observed in hybrid zones, where early‐life survivorship favors certain genotypes over others (Lexer et al. 2005) and can also result in the preferential retention of alleles that confer fitness advantages. For example, specific loci linked to stress tolerance—such as MYB transcription factors—can provide a selective advantage to certain hybrid genotypes, in this case particularly those with a greater proportion of *P. alba* ancestry (Bresadola et al. 2019).

Previous studies of natural populations in Austria, Hungary, and Italy (Bresadola et al. 2019; Lexer et al. 2010; van Loo et al. 2008) suggest that backcrosses to *P. alba* are the most prevalent in natural hybrid zones. However, the studies by Lindtke et al. (2014) and Christe et al. (2016) in these hybrid zones, together with Santos‐del‐Blanco et al. (2013) on the Iberian Peninsula, indicated that F_1_ hybrids are the most common genotypes in nature. Nevertheless, natural hybrid populations consistently show similar patterns: low number of adult individuals or lack of the parental *P. tremula* and its backcrosses. This could be explained partially by the above‐mentioned genetic mechanisms contributing to the asymmetric postzygotic selection, favoring *P. alba* ‐like genotypes, as also observed in our common garden experiment.

It is important to note that the examined survivorship of saplings from sexual reproduction is less critical within already established natural hybrid zones, where vegetative propagation can sustain populations, but propagation via seeds is likely to play a central role in colonizing new habitats (Macaya‐Sanz et al. 2016; Vallejo‐Marín et al. 2010).

### 4.2 Rooting Advantage Comes From *P. alba* Ancestry

Despite hybrid origin, *P. × canescens* is diploid, remains fully sexual and produces viable seeds. However, this natural hybrid is often maintained locally by clonal regeneration (He et al. 2010; Macaya‐Sanz et al. 2016; Santos‐del‐Blanco et al. 2013; van Loo et al. 2008). Although seed fertility is not inherently limited, successful sexual reproduction can encounter several ecological constraints. *Populus* species´ seeds are in general short‐lived, require continuously moist substrate conditions for germination, and are highly sensitive to water table fluctuations and soil moisture deficits (Guilloy‐Froget et al. 2002; Stella et al. 2006). Consequently, seeds rarely lead to sustained seedling recruitment unless conditions are ideal, typically following habitat disturbances such as flooding or land clearing (Braatne et al. 1996; Stella et al. 2006). The absence of such specific habitats suggests that vigorous hybrid clones persist primarily through root suckering, even if contemporary hybridization events become rare. *P. × canescens* hybrids tend to form larger clones over extensive areas compared to *P. alba* as reported by van Loo et al. (2008). Although other hybrid zone studies suggest that clonality is more pronounced in *P. alba* compared to the hybrid (Santos‐del‐Blanco et al. 2013), *P. × canescens* and especially backcrosses clone at least as readily as *P. alba* (van Loo et al. 2008).

In this study, clonality was quantified as the survival of shoot cuttings. We emphasize that this metric does not measure natural clonal establishment via root suckering in situ. Rather, it provides a management‐relevant proxy for the ability to generate viable clonal planting material in nursery‐like settings, which is the primary pathway through which *Populus* clones are propagated for forestry and restoration. At the same time, shoot‐rooting capacity can retain ecological relevance because stem fragments produced by storms or floods may be transported downstream and root on freshly deposited alluvium, as documented for *P. nigra* (Guilloy‐Froget et al. 2002; Rood et al. 2003) and suggested for *P. alba* and *P. × canescens* (Dickmann and Kuzovkina 2014; Macaya‐Sanz et al. 2016). Identifying genotypes with high shoot‐rooting capacity may therefore aid both clonal forestry and riparian restoration, and recent nursery trials likewise report substantial among‐genotype variation in shoot‐rooting performance within *P. × canescens* (Pokorná et al. 2024). Our data show that genetic ancestry shapes clonability in the *P. alba × P. tremula* complex. Among the material we tested, cuttings whose genomes were biased toward *P. alba* rooted and survived best, which aligns well with earlier reports showing that *P. alba* is inherently easier to root than aspens and most other members of the *Populus* section. Bannoud and Bellini (2021) report that mature, woody cuttings from most species in the *Populus section* seldom form adventitious roots. A few *P. alba* clones are an exception and still root readily. Aspens such as *P. tremula* root only when soft, current‐season shoots are used, and even then, they require costly greenhouse‐misting conditions. Temporal analyses corroborate this difference: Güneş (2000) observed that both *P. alba* and *P. tremula* initiate root primordia ≈10 days after excision, yet elongation proceeds only in *P. alba* ; Chen et al. (1994) found that *P. alba* rooted within 14 days, while the aspen representative *P. davidiana* produced no roots at all. Building on those species‐level contrasts, Dickmann et al. (2001) showed that a subset of *P. alba* genotypes even roots from dormant lignified shoot segments, underscoring the genetic component behind this trait as well. Our present experiment mirrors this gradient exactly: rooting success was highest in pure *P. alba* and in backcross hybrids carrying more *P. alba* alleles, intermediate in the balanced *P. tremula × P. alba* material, and lowest in the pure *P. tremula* class. Such additive inheritance patterns are expected; clone‐level studies across 21 *Populus* taxa likewise reported that rooting traits partition between and within pedigree groups, giving hybrids intermediate values (Zalesny Jr. et al. 2005). Furthermore, the interaction we detected between germination year and genotype, where older stock rooted more poorly, fits aforementioned observations. Work on dormant lignified shoot cuttings has shown that shoot age/position and the date of collection depress rooting percentages as tissues become more mature (Zalesny Jr. et al. 2003; Zalesny Jr. and Wiese 2006). Our data therefore reinforces the view that both genetic background and the physiological age of donor material act synergistically to determine clonal propagation success in *Populus*.

### 4.3 *Populus alba* Ancestry Elevates Juvenile Growth in Root‐Sucker Poplars

In clonal *Populus* populations, early stand development is driven mainly by the elongation of root suckers; therefore, our growth analysis deliberately centered on root‐cutting saplings. This propagation path mirrors both natural vegetative spread and current nursery practice, so the results speak directly to breeding, conservation, and restoration programs that deploy clones selected in the field. Although physiological differences mean that root‐sourced ramets are not strictly comparable to seed‐originated seedlings, they constitute the most ecologically and silviculturally relevant benchmark for evaluating juvenile performance in hybrid poplars.

Within this framework, we found that root‐cutting saplings with greater *P. alba* ancestry were significantly taller at the end of the 2018 growing season than those biased toward *P. tremula* . True head‐to‐head data on juvenile growth of European aspen versus white poplar are scarce, but our findings imply that *P. alba* possesses and transmits greater early height potential. Paradoxically, in our trial, the pure *P. alba* saplings recorded a lower relative growth rate (RGR) over the 2018 season than *P. tremula* . This does not mean they grew less in absolute terms; *P. alba* seedlings started the season taller, so much of their final height was carried over from the previous year, making their proportional gain in 2018 appear smaller. Because these low *P. alba* RGR values masked broader patterns, our initial mixed models (which pooled all genotypes) failed to detect a significant RGR trend. Removing the pure parental classes clarified the picture: among hybrids, increasing *P. alba* admixture was positively correlated with RGR, pointing to additive or partially dominant effects of *P. alba* alleles on juvenile vigor.

Hybrid vigor is a recurrent theme in the genus. In other *Populus* groups, F_1_ or backcross hybrids outgrow their parents in volume increment, wood yield, or stress tolerance (Ceulemans et al. 1992; Scaracia‐Mugnozza et al. 1997; Zanewich et al. 2018). Maternal versus paternal contributions can modulate that advantage. For example, in *P. alba × P. euphratica* hybrids the combination with *P. alba* as the seed parent grew fastest (Sohrabi et al. 2015). The maternal origin of our *P. tremula × P. alba* hybrids remains, however, unknown. Also, *P. × canescens* did not greatly outperform both parents across the board. Long‐term growth and stress resilience with relation to the sex still warrant dedicated study.

The negative impact of the germination year on height further suggests that older propagation material may possess diminished growth potential, a trend that has also been observed when analyzing clonality. Furthermore, the significant variation in growth observed across different blocks within the common garden underscores the role of micro‐environmental conditions in modulating genetic potential. Local growing conditions can significantly influence the performance of poplars.

Our study provides robust evidence that traits derived from *P. alba* confer distinct advantages in juvenile growth. These findings have important implications for breeding programs and conservation strategies, suggesting that selecting for *P. alba* traits in hybrids could optimize early growth performance. Since growth strongly impacts competition, carbon sequestration, and climate adaptation, selecting high‐performing hybrids could improve reforestation efforts, afforestation programs, and sustainable forestry (Adler et al. 2021; Houminer et al. 2024; Reed‐Métayer et al. 2025; Sunarti and Nirsatmanto 2021).

### 4.4 *Populus alba* Admixture Governs Pubescence

Leaf pubescence is a classic quantitative trait: it exhibits continuous variation, is highly heritable, and is controlled by a compact yet hierarchically organized gene network (Bloomer et al. 2014; Mauricio 2005; Pattanaik et al. 2014). In poplars, small changes in the copy number, expression level or epigenetic control of key regulatory loci are sufficient to toggle the phenotype from virtually glabrous to densely hairy, with cascading effects on photosynthesis, water relations and defence (Bewg et al. 2022; Plett et al. 2010). Several lines of evidence point to the MYB–bHLH–WD40 (MBW) gene complex as the key switch for nonglandular trichome formation. When the MYB gene is over‐expressed in *P. tremula* × *P. alba* , leaves grow more hairs (Plett et al. 2010). The opposite is also true: CRISPR/Cas9 lines that successively lost one, two, or all MYB copies produced fewer and fewer hairs until completely smooth leaves appeared (Bewg et al. 2022). Another way of control comes from the m^6^A RNA‐methyltransferase (MTA), which tags MBW transcripts with N^6^‐methyladenosine marks determining a further increase in hair density (Lu et al. 2020).

Taken together, these studies indicate that alterations in the regulatory landscape, whether via copy‐number variation, shifts in transcriptional activity, or epigenetic modulation, can recalibrate leaf pubescence in hybrids. Our common‐garden trial corroborates this inference: leaf pubescence, alongside survivorship, clonality, and growth, stands out as the fourth functional trait whose expression reflects underlying genomic composition. Abaxial reflectance (a proxy for hairiness) increased with the fraction of *P. alba* ancestry and followed a significant quadratic relationship with the hybrid index *q* (Figure 5a). The pronounced curvature indicates that *P. alba* and its backcrosses carry the densest pubescence, F_1_ hybrids are intermediate, and *P. tremula* along with its backcrosses remain essentially glabrous. Many recombinant F_n_ hybrids, however, fell below this curve, implying that meiotic shuffling disrupted epistatic gene combinations required for dense pubescence; breaking up these coadapted alleles lowers hair density, a pattern previously reported for other *Populus* traits (Bresadola et al. 2019). Similar epistatic effects can be encountered across other plants: in species as different as *Arabidopsis* (Bloomer et al. 2014) or soybean (Liu et al. 2020), dense leaf or fibre hairs appear only when the right allele combinations are maintained; once those combinations are broken, pubescence decreases sharply. These cross‐species parallels underline how crucial intact gene networks are for maintaining hairiness.

As regulatory modulation of genes controlling pubescence defines the genetic potential for trichome production and realized trichome density scales predictably with the proportion of *P. alba* alleles, the question arises whether the resulting hair gradient aligns with plant performance. Dense trichomes are known to moderate leaf level stresses by cooling the lamina, limiting transpiration, scattering UVB, and impeding sapsucking insects (Karabourniotis et al. 2020). Elevating MYB dosage, especially when reinforced by m^6^A tagging via MTA, packs leaves with more trichomes and triterpenes, strengthens the cuticle, curbs water loss, deters pests, and simultaneously promotes faster seedling growth, better rooting, and greater drought tolerance (Plett et al. 2010; Liu et al. 2024; Lu et al. 2020). In our common garden experiment, we observed a similar trend: genotypes with denser leaf trichomes appeared to grow taller, root more successfully from cuttings, and survive their first years in higher numbers, whereas sparsely pubescent, *P. tremula* ‐biased genotypes tended to lag behind. While we did not statistically test these associations, the parallel patterns observed across survival, growth, and pubescence suggest that trichome density may be one of several *P. alba* ‐derived traits that covary with enhanced early‐life performance in hybrid poplars.

### 4.5 Conclusion: Practical Implications for Conservation and Applied Forestry

Collectively, our findings on survivorship, clonality, growth, and leaf pubescence highlight a cohesive adaptive suite driven largely by genetic ancestry biased towards *P. alba* . Genotypes with higher proportions of *P. alba* ancestry and F_1_ hybrids exhibited superior early‐life survival, significantly improved rooting capacity, enhanced juvenile growth rates, and increased leaf pubescence. This adaptive combination is particularly advantageous in disturbed, drought‐prone, or harsh environments such as floodplains or degraded riparian zones, where rapid establishment, efficient resource use, and stress resilience are critical for successful forest restoration and establishment.

Given the strong heritability and straightforward genetic basis of trichome density observed in our study, pubescence emerges as a promising trait for targeted breeding or even genomic editing efforts aimed at enhancing drought tolerance, pest resistance, and overall stress mitigation without compromising growth rates. Such efforts are especially relevant in urban forestry contexts, restoration projects in ecologically vulnerable riparian habitats, and in short‐rotation biomass production systems where rapid growth, resilience, and environmental adaptability are crucial. Building on the leaf‐reflectance protocol introduced by Lexer et al. (2009), we adopted the method and demonstrated its practicality as a proxy for assessing genetic admixture in hybrid populations. This trait‐based approach enables swift screening of nursery stock or wild stands, helping identify genotypes best suited to specific site conditions while avoiding the time and expense of genomic analyses. By showcasing its use in real‐world settings, we further recommend integrating reflectance screening into breeding and restoration programs to streamline selection and enhance operational efficiency.

While the precise long‐term advantages of hybrid genotypes remain only partly resolved, particularly with respect to performance across multiple seasons and under chronic stress, our initial findings point to considerable potential. The observed decline in fitness of recombinant hybrids over successive generations underscores the need for strategic planting schemes that prioritize either pure *P. alba* or backcrosses and F_1_ hybrids, wherever superior growth and stress tolerance are required. Even so, pure *P. tremula* continues to play an irreplaceable ecological role by supporting biodiversity, sustaining trophic networks and contributing genetic resilience. Therefore, it must remain an integral component of conservation and forestry programs. Nursery propagation of *P. tremula* involves practical challenges: seed‐based production carries lower early‐stage survivorship, and stem cuttings root at modest rates, meaning propagators should start with a larger pool of material to reach target numbers. When clonal stock is needed, root cuttings still provide the most dependable route for mass‐producing vigorous saplings suitable for reforestation and restoration.

In conclusion, the practical recommendations provided in our study emphasize the considerable potential of *P. alba* ‐influenced hybrids for forestry applications and ecological restoration. Leveraging genetic insights to select optimal hybrid and pure‐species genotypes enables forest managers and conservationists to maximize both ecological and economic outcomes, thereby.

## Funding

This work was supported by grant 31003A_149306 from the Swiss National Science Foundation and the professorship start‐up grant BE772002 at the University of Vienna (both awarded to Ch. Lexer), as well as by the Fondazione Bussolera Branca (Mairano, Pavia, Italy) within the framework of the research project “Biodiversity of Natural and Cultivated Poplar.” The latter also facilitated the establishment of the common garden in Italy at “Vivai Piante Luigi Gambardella” in Mercato San Severino (Salerno, Italy). This research was also funded in part by the Austrian Science Fund (FWF) (DOI: https://doi.org/10.55776/T416). For open access purposes, the author has applied a CC BY public copyright license to any author accepted manuscript version arising from this submission.

## Conflicts of Interest

The authors declare no conflicts of interest.

## Supporting information


**Table S1:** Fixed coefficients of the best fitted GLMM for the third year survivorship.
**Table S2:** Model comparison and χ^2^‐test between the five GLMMs of seedling survivorship. In each model, maternal family was introduced as a random factor. Estimates of fixed coefficients evaluation of each model are added to the predictors.
**Table S3:** Model comparison and χ^2^‐test between the five GLMMs of clonability. Germination year of propagation source was considered as a random factor in the models. Estimates of fixed coefficients evaluation of each model are added to the predictors.
**Table S4:** Fixed coefficients of the best fitted model of cutting success.
**Table S5:** Model comparison and χ^2^‐test between the eight height growth LMMs. Included height measurements of each month of all trees as a response variable, April was used as baseline for measurement time points. In each LMM, location in the CG (block number) was added to the models as a random factor. Values of fixed coefficients evaluation of each model are added to the predictors.
**Table S6:** Significance of fixed coefficients of best fitting model using height measurements of each month as a responds variable.
**Table S7:** Model comparison and χ^2^‐test between the eight RGR LMMs, RGR calculated for each month with April as reference. In each LMM, location in the CG (block number) was added to the models as a random factor. Values of fixed coefficients evaluation of each model are added to the predictors.
**Table S8:** Model comparison and χ^2^‐test between the RGR LMMs, RGR was calculated for whole the vegetation period of 2018, location in the CG (block number) was added to the LMMs as a random factor. Values of fixed coefficients evaluation of each model are added to the predictors.
**Table S9:** Model comparison and χ^2^‐test between the RGR of hybrids LMMs, RGR was calculated for whole the vegetation period of 2018, location in the CG (block number) was added to the LMMs as a random factor. Values of fixed coefficients evaluation of each model are added to the predictors.
**Table S10:** Significance of fixed coefficients of the top‐rank model using only overall RGR of hybrids as a response variable.
**Table S11:** Pearson correlation coefficients (*r*) among three variables related to leaf reflectance: Mean Grey Value, Modal Grey Value, and Median Grey Value.
**Table S12:** Model selection using ANOVA of mixed models during the analyses of leaf reflectance and genetic ancestry parameters.
**Table S13:** Analysis of variance and F test between the four linear models of leaf reflectance. Estimates of fixed coefficients evaluation of each model are added to the predictors.
**Table S14:** ANOVA and F test between our linear models for analysing genetic parameters and abaxial surface hairiness.
**Table S15:** Significance of fixed coefficients of the top‐rank linear model (*y ~ x + x* ^*2*^) in case of investigating leaf reflectance and genetic ancestry parameters' relationship.
**Figure S1:** Walter‐and‐Lieth climate diagrams for the two common‐garden sites, CG‐Swiss (A) and CG‐Italy (B), over the experimental period 2010–2017. Monthly total precipitation is shown as blue vertical bars (right‐hand y‐axis, mm) and mean monthly air temperature as a red line (left‐hand y‐axis, °C). Data are from the E‐OBS v23.1 gridded dataset (Cornes et al. 2018) accessed via the Copernicus Surf‐OBS portal, and the plots were produced with the *climatol* R package (diagwl function). When monthly precipitation is greater than 100 mm, the scale is increased from 2 mm/C to 20 mm/C to avoid too high diagrams in very wet locations. This change is indicated by a black horizontal line, and the graph over it is filled in solid blue. When the precipitation graph lies under the temperature graph (*p* < 2 T) we have an arid period (filled in dotted red vertical lines), otherwise the period is considered wet (filled in blue lines). The blue rectangles for each month on the x‐axis indicate the likelihood of frost days. When the average daily minimum is zero or negative, frost certainly occurs and the rectangle is filled with dark blue. If it is zero or positive, the rectangle is filled with a lighter blue to indicate the probability of having frosts in that month. White rectangles indicate months with no frost days.
**Figure S2:** Triangle plot of survivorship in the third year according to *q* (x‐axis) and Q_12_ (y‐axis) values in Fribourg in CG‐Swiss (on the left) and in Salerno in CG‐Italy (on the right).
**Figure S3:** Predicted probability curves for each germination year of cutting success along the scale of *q* with 95% confidence intervals.
**Figure S4:** Polynomial regression line for the same model shown separately by genotype, representing changes in height during the six measurement time points.
**Figure S5:** Scatterplot with 95% confidence interval of the best fitting model for height changes in each months, showing the effects of blocks on height.
**Figure S6:** Plot of Spearman's rank test shows a positive correlation between RGR calculated from height data and RGR from diameter.
**Figure S7:** Relationship between *q* and overall RGR calculated for 2018.
**Figure S8:** Regression line of the top rank model for overall RGR of hybrids showing effects of blocks in the CG.

## Data Availability

Data supporting this study is publically available in the Dryad Digital Repository: https://doi.org/10.5061/dryad.sj3tx96k8
