# Supplementary material for: Effect of Genetic Ancestry on Ecologically Important Fitness Traits in Hybridizing Populus Species: Relevance for Conservation and Forest Management
Source: Evol Appl. 2026 Mar 25;19(3):e70215. doi: 10.1111/eva.70215 (PMC13093791; doi:10.1111/eva.70215)
Supplement: Supplementary file 1 — Table S1: Fixed coefficients of the best fitted GLMM for the third year survivorship. Table S2: Model comparison and χ2‐test between the five GLMMs of seedling survivorship. In each model, maternal family was introduced as a random factor. Estimates of fixed coefficients evaluation of each model are added to the predictors. Table S3: Model comparison and χ2‐test between the five GLMMs of clonability. Germination year of propagation source was considered as a random factor in the models. Estimates of fixed coefficients evaluation of each model are added to the predictors. Table S4: Fixed coefficients of the best fitted model of cutting success. Table S5: Model comparison and χ2‐test between the eight height growth LMMs. Included height measurements of each month of all trees as a response variable, April was used as baseline for measurement time points. In each LMM, location in the CG (block number) was added to the models as a random factor. Values of fixed coefficients evaluation of each model are added to the predictors. Table S6: Significance of fixed coefficients of best fitting model using height measurements of each month as a responds variable. Table S7: Model comparison and χ2‐test between the eight RGR LMMs, RGR calculated for each month with April as reference. In each LMM, location in the CG (block number) was added to the models as a random factor. Values of fixed coefficients evaluation of each model are added to the predictors. Table S8: Model comparison and χ2‐test between the RGR LMMs, RGR was calculated for whole the vegetation period of 2018, location in the CG (block number) was added to the LMMs as a random factor. Values of fixed coefficients evaluation of each model are added to the predictors. Table S9: Model comparison and χ2‐test between the RGR of hybrids LMMs, RGR was calculated for whole the vegetation period of 2018, location in the CG (block number) was added to the LMMs as a random factor. Values of fixed coefficients evaluation of eac [file EVA-19-e70215-s001.docx]

**Supplementary Materials**

**1. Supplementary Tables**

Table S1. Fixed coefficients of the best fitted GLMM for the 3^rd^ year survivorship.

|  | Estimate | Std..Error | z.value | Pr(>\|z\|) |
| --- | --- | --- | --- | --- |
| Intercept | 1.799 | 0.432 | 4.165 | <0.0001 |
| q | -31.334 | 6.372 | -4.917 | <0.0001 |
| q^2^ | 32.630 | 6.350 | 5.138 | <0.0001 |
| Q_12_ | 16.777 | 3.825 | 4.386 | <0.0001 |

Table S2. Model comparison and Chi^2^-test between the five GLMMs of seedling survivorship. In each model, maternal family was introduced as a random factor. Estimates of fixed coefficients evaluation of each model are added to the predictors.

Model legend:

GLMM1: glmm with the random intercept that varies across families;

GLMM2: added the fixed effect of the *q*;

GLMM3: added the fixed effect of the *Q*_12_;

GLMM4: added the fixed effect of the *q*^2^;

GLMM5: added the fixed effect of the *Q*^2^_12_;

| Model | Intercept | q linear | q quad | Q12 linear | CG | df | logLik | AIC | delta AIC | p.value |
| --- | --- | --- | --- | --- | --- | --- | --- | --- | --- | --- |
| GLMM1 | 2.91 |  |  |  |  | 2 | -64.7 | 133.5 |  |  |
| GLMM2 | 1.34 | 3.95 |  |  |  | 3 | -60.1 | 126.1 | 7.4 | 0.0022 |
| GLMM3 | 2.04 | -3.55 | 9.13 |  |  | 4 | -58.4 | 124.7 | 1.4 | 0.0652 |
| GLMM4 | 1.80 | -31.33 | 32.63 | 16.78 |  | 5 | -46.4 | 102.7 | 22.0 | <0.0001 |
| GLMM5 | 1.29 | -31.57 | 33.19 | 16.94 | 1.05 | 6 | -44.7 | 101.4 | 1.4 | 0.0672 |

Table S3. Model comparison and Chi^2^-test between the five GLMMs of clonability. Germination year of propagation source was considered as a random factor in the models. Estimates of fixed coefficients evaluation of each model are added to the predictors.

GLMM1: a model in which the random intercept varies for every germination year;

GLMM 2: a model with the predictor *q* inserted as fixed effect and still the random intercept;

GLMM3: same as above but with also the *Q*_12_ as fixed effect;

GLMM4: to the GLMM3 has been added also the fixed effect of the diameter class as a covariate.

| Model | Intercept | q | Q12 | DC | df | logLik | AIC | delta AIC | p.value |
| --- | --- | --- | --- | --- | --- | --- | --- | --- | --- |
| GLMM1 | -0.56 |  |  |  | 2 | -700.6 | 1405.1 |  |  |
| GLMM2 | -1.90 | 1.84 |  |  | 3 | -655.4 | 1316.8 | 88.4 | <0.0001 |
| GLMM3 | -1.68 | 1.69 | -0.25 |  | 4 | -654.3 | 1316.6 | 0.1 | 0.1428 |
| GLMM4 | -1.61 | 1.70 | -0.25 | -0.03 | 5 | -654.1 | 1318.2 | -1.6 | 0.5348 |

Table S4. Fixed coefficients of the best fitted model of cutting success

|  | Estimate | Std. Error | z.value | p.value |
| --- | --- | --- | --- | --- |
| Intercept | -1.90166 | 0.232032 | -8.19569 | <0.0001 |
| q | 1.836514 | 0.205571 | 8.933744 | <0.0001 |

Table S5. Model comparison and Chi^2^-test between the eight height growth LMMs. Included height measurements of each month of all trees as a response variable, April was used as baseline for measurement time points. In each LMM, location in the CG (block number) was added to the models as a random factor. Values of fixed coefficients evaluation of each model are added to the predictors.

Model legend:

GLS1: model with only the intercept;

LME1: the random intercept varies between the blocks;

LME2: the variable “Time” is included as fixed effect;

LME3: the quadratic effect of time is inserted as fixed effect;

LME4: the cubic effect of time is inserted as fixed effect;

LME5: to the previous model is inserted the fixed effect of the *q*;

LME6: added the fixed effect of the germination year;

LME7: finally also the fixed effect of the *Q_12_* was evaluated.

| Model | Intercept | time linear | time quad | time cubic | q linear | Q12 linear | GY | df | logLik | AIC | delta AIC | p.value |
| --- | --- | --- | --- | --- | --- | --- | --- | --- | --- | --- | --- | --- |
| GLS1 | 205.42 |  |  |  |  |  |  | 2 | -5687.1 | 11378.2 |  |  |
| LME1 | 205.35 |  |  |  |  |  |  | 3 | -5678.7 | 11363.5 |  |  |
| LME2 | 163.55 | 11.94 |  |  |  |  |  | 4 | -5553.6 | 11115.2 | 248.3 | <0.0001 |
| LME3 | 162.77 | 12.53 | -0.08 |  |  |  |  | 5 | -5553.6 | 11117.1 | -2.0 | 0.8625 |
| LME4 | 192.19 | -24.49 | 12.18 | -1.17 |  |  |  | 6 | -5548.6 | 11109.1 | 8.0 | 0.0015 |
| LME5 | 173.62 | -24.49 | 12.18 | -1.17 | 27.39 |  |  | 7 | -5536.0 | 11086.0 | 23.1 | <0.0001 |
| LME6 | 5206.76 | -24.49 | 12.18 | -1.17 | 31.53 |  | -2.50 | 8 | -5530.5 | 11076.9 | 9.1 | 0.0009 |
| LME7 | 5005.07 | -24.49 | 12.18 | -1.17 | 30.05 | -6.56 | -2.40 | 9 | -5529.6 | 11077.1 | -0.2 | 0.1790 |

Table S6. Significance of fixed coefficients of best fitting model using height measurements of each month as a responds variable

|  | Value | Std.Error | DF | t.value | p.value |
| --- | --- | --- | --- | --- | --- |
| Intercept | 5206.755 | 1511.047 | 1076 | 3.445794 | 0.0006 |
| Month | -24.4884 | 12.00477 | 1076 | -2.03989 | 0.0416 |
| I(Month^2) | 12.178 | 3.841469 | 1076 | 3.170142 | 0.0016 |
| I(Month^3) | -1.16784 | 0.363008 | 1076 | -3.21713 | 0.0013 |
| q | 31.5313 | 5.561592 | 1076 | 5.669473 | <0.0001 |
| GY | -2.50248 | 0.75127 | 1076 | -3.33099 | 0.0009 |

Table S7. Model comparison and Chi^2^-test between the eight RGR LMMs, RGR calculated for each month with April as reference. In each LMM, location in the CG (block number) was added to the models as a random factor. Values of fixed coefficients evaluation of each model are added to the predictors.

Model legend:

GLS1: model with only the intercept;

LME1: the random intercept varies between the blocks;

LME2: the variable “Time” is included as fixed effect;

LME3: the quadratic effect of time is inserted as fixed effect;

LME4: the cubic effect of time is inserted as fixed effect;

LME5: to the previous model is inserted the fixed effect of the *q*;

LME6: added the fixed effect of the germination year;

LME7: finally also the fixed effect of the *Q_12_* was evaluated.

| Model | Intercept | time linear | time quad | time cubic | q linear | Q12 linear | GY | df | logLik | AIC | delta AIC | p.value |
| --- | --- | --- | --- | --- | --- | --- | --- | --- | --- | --- | --- | --- |
| GLS1 | 0.000952 |  |  |  |  |  |  | 2 | 5146.7 | -10289.3 |  |  |
| LME1 | 0.000949 |  |  |  |  |  |  | 3 | 5175.8 | -10345.7 | 56.3 | <0.0001 |
| LME2 | -0.000095 | 0.000348 |  |  |  |  |  | 4 | 5399.8 | -10791.5 | 445.8 | <0.0001 |
| LME3 | -0.000439 | 0.000643 | -0.000049 |  |  |  |  | 5 | 5407.9 | -10805.8 | 14.3 | 0.0001 |
| LME4 | 0.000406 | -0.000545 | 0.000404 | -0.00005 |  |  |  | 6 | 5416.9 | -10821.8 | 16 | <0.0001 |
| LME5 | 0.000427 | -0.000545 | 0.000404 | -0.00005 | -0.000031 |  |  | 7 | 5417 | -10819.9 | -1.9 | 0.7344 |
| LME6 | -0.023056 | -0.000545 | 0.000404 | -0.00005 | -0.00005 |  | 0.000012 | 8 | 5417.4 | -10818.8 | -1.1 | 0.3549 |
| LME7 | -0.020571 | -0.000545 | 0.000404 | -0.00005 | -0.000032 | 0.000081 | 0.00001 | 9 | 5417.9 | -10817.7 | -1 | 0.3261 |

Table S8. Model comparison and Chi^2^-test between the RGR LMMs, RGR was calculated for whole the vegetation period of 2018, location in the CG (block number) was added to the LMMs as a random factor. Values of fixed coefficients evaluation of each model are added to the predictors.

Model legend:

GLS1: model with only the intercept;

LME1: the random intercept varies between the blocks;

LME2: the variable *q* is included as fixed effect;

LME3: the Germination Year is included as fixed effect;

LME4: the variable *Q*12 is included as fixed effect;

| Model | Intercept | q linear | Q12 linear | GY | df | logLik | AIC | delta AIC | p.value |
| --- | --- | --- | --- | --- | --- | --- | --- | --- | --- |
| GLS1 | 0.0014938 |  |  |  | 2 | 1023.6 | -2043.2 |  |  |
| LME1 | 0.0003748 |  |  |  | 3 | 1037.6 | -2069.2 | 26.1 | <0.0001 |
| LME2 | 0.0014579 | 0.0000449 |  |  | 4 | 1037.6 | -2067.3 | -2.0 | 0.8609 |
| LME3 | -0.0291600 | 0.0000198 |  | 0.0000152 | 5 | 1037.7 | -2065.5 | -1.8 | 0.6683 |
| LME4 | -0.0263138 | 0.0000406 | 0.0000924 | 0.0000138 | 6 | 1037.8 | -2063.6 | -1.8 | 0.6896 |

Table S9. Model comparison and Chi^2^-test between the RGR of hybrids LMMs, RGR was calculated for whole the vegetation period of 2018, location in the CG (block number) was added to the LMMs as a random factor. Values of fixed coefficients evaluation of each model are added to the predictors.

Model legend:

GLS1: model with only the intercept;

LME1: the random intercept varies between the blocks;

LME2: the variable *q* is included as fixed effect;

LME3: the Germination Year is included as fixed effect;

LME4: the variable *Q*12 is included as fixed effect;

| Model | Intercept | q linear | Q12 linear | GY | df | logLik | AIC | delta AIC | p.value |
| --- | --- | --- | --- | --- | --- | --- | --- | --- | --- |
| GLS1 | 0.00151 |  |  |  | 2 | 868.7 | -1733.4 |  |  |
| LME1 | 0.00151 |  |  |  | 3 | 880.8 | -1755.6 | 22.2 | <0.0001 |
| LME2 | 0.00073 | 0.00116 |  |  | 4 | 884.5 | -1761.0 | 5.4 | 0.0067 |
| LME3 | -0.08603 | 0.00113 |  | 0.00004 | 5 | 885.1 | -1760.3 | -0.7 | 0.2468 |
| LME4 | -0.08540 | 0.00113 | 0.00002 | 0.00004 | 6 | 885.1 | -1758.3 | -2.0 | 0.9667 |

Table S10. Significance of fixed coefficients of the top-rank model using only overall RGR of hybrids as a response variable

|  | Value | Std.Error | DF | t.value | p.value |
| --- | --- | --- | --- | --- | --- |
| Intercept | 0.000734 | 0.00034 | 148 | 2.159 | 0.0324 |
| q | 0.001156 | 0.000424 | 148 | 2.727 | 0.0071 |

Table S11. Pearson correlation coefficients (r) among three variables related to leaf reflectance: Mean Gray Value, Modal Gray Value, and Median Gray Value

|  | Mean Gray | Modal Gray | Median Gray |
| --- | --- | --- | --- |
| Mean Gray | 1 | 0,993706 | 0,999529 |
| Modal Gray | 0,993706 | 1 | 0,995669 |
| Median Gray | 0,999529 | 0,995669 | 1 |

Table S12. Model selection using ANOVA of mixed models during the analyses of leaf reflectance and genetic ancestry parameters

Model legend:

GLS1: a linear model with only the intercept;

LME1: a linear mixed effect model in which the random intercept varies for each block;

| Model | df | AIC | delta AIC | BIC | logLik | Test | L.Ratio | *p*-value |
| --- | --- | --- | --- | --- | --- | --- | --- | --- |
| GLS1 | 2,0 | 1868,7 |  | 1875,3 | -932,4 |  |  |  |
| LME1 | 3,0 | 1870,5 | -1,8 | 1880,4 | -932,3 | 1 vs 2 | 0,2 | 0,6465 |

Table S13. Analysis of variance and F test between the four linear models of leaf reflectance. Estimates of fixed coefficients evaluation of each model are added to the predictors.

Model legend:

LM1: a linear model with only the intercept;

LM2: a linear model with the predictor *q*;

LM3: in this model have been evaluated the effects of the *q* in addition to the effect of the *q*^2^;

LM4: to the model has been added also the effect of the *Q*_12_ index.

| Model | Intercept | q linear | q quad | Q12 linear | df | RSS | delta RSS | p.value |
| --- | --- | --- | --- | --- | --- | --- | --- | --- |
| LM1 | 161.05 |  |  |  |  | 142654.3 |  |  |
| LM2 | 94.80 | 97.88 |  |  | 1 | 49861.2 | 92793.1 | <0.0001 |
| LM3 | 102.26 | 58.70 | 37.56 |  | 1 | 48273.3 | 1587.9 | 0.0123 |
| LM4 | 102.23 | 36.38 | 60.31 | 7.50 | 1 | 48198.9 | 74.3 | 0.5850 |

Table S14. ANOVA and F test between our linear models for analysing genetic parameters and abaxial surface hairiness

| Predictors | Df | SS | RSS | F | p.value |
| --- | --- | --- | --- | --- | --- |
| Intercept | NA | NA | 142654.31 | NA | NA |
| q | 1 | 92793.099 | 49861.21 | 373.4907 | <0.0001 |
| q+q^2^ | 1 | 1587.918 | 48273.29 | 6.391344 | 0.0123 |
| q+q^2^+Q_12_ | 1 | 74.347 | 48198.95 | 0.299243 | 0.5850 |

Table S15. Significance of fixed coefficients of the top-rank linear model (*y ~ x + x²)* in case of investigating leaf reflectance and genetic ancestry parameters´ relationship

| Predictors | Estimate | Std..Error | t.value | Pr(>\|t\|) |
| --- | --- | --- | --- | --- |
| Intercept | 102.2989 | 4.64277 | 22.03401 | <0.0001 |
| q | 58.6453 | 16.21729 | 3.61622 | 0.000381 |
| q^2^ | 37.4115 | 14.77157 | 2.53267 | 0.012108 |

**2. Supplementary Figures**

Figure S1. Walter-and-Lieth climate diagrams for the two common-garden sites, CG-Swiss (A) and CG-Italy (B), over the experimental period 2010–2017. Monthly total precipitation is shown as blue vertical bars (right-hand y-axis, mm) and mean monthly air temperature as a red line (left-hand y-axis, °C). Data are from the E-OBS v23.1 gridded dataset (Cornes et al., 2018) accessed via the Copernicus Surf-OBS portal, and the plots were produced with the *climatol* R package (diagwl function). When monthly precipitation is greater than 100 mm, the scale is increased from 2 mm/C to 20 mm/C to avoid too high diagrams in very wet locations. This change is indicated by a black horizontal line, and the graph over it is filled in solid blue. When the precipitation graph lies under the temperature graph (P < 2T) we have an arid period (filled in dotted red vertical lines), otherwise the period is considered wet (filled in blue lines). The blue rectangles for each month on the x-axis indicate the likelihood of frost days. When the average daily minimum is zero or negative, frost certainly occurs and the rectangle is filled with dark blue. If it is zero or positive, the rectangle is filled with a lighter blue to indicate the probability of having frosts in that month. White rectangles indicate months with no frost days.


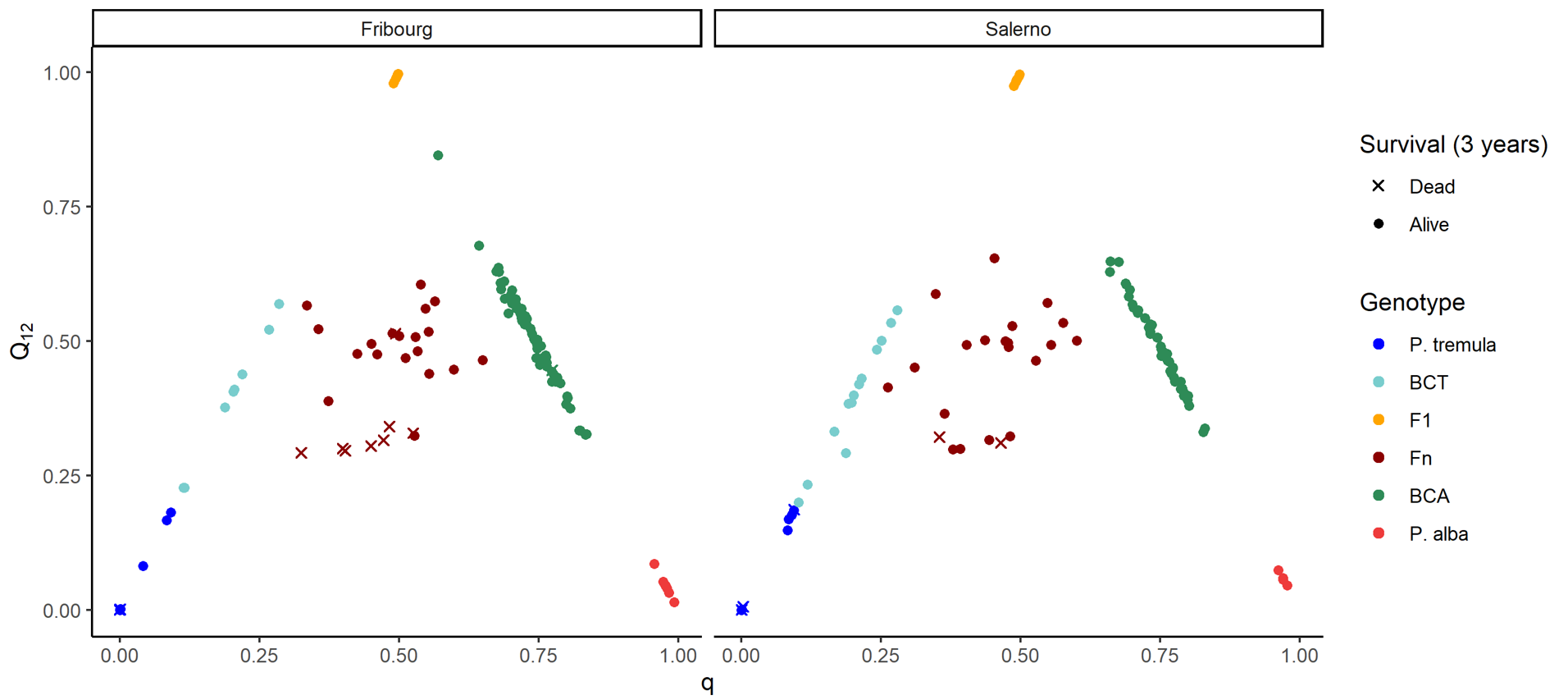


Figure S2. Triangle plot of survivorship in the third year according to *q* (x-axis) and Q_12_ (y-axis) values in Fribourg in CG-Swiss ( on the left) and in Salerno in CG-Italy (on the right).


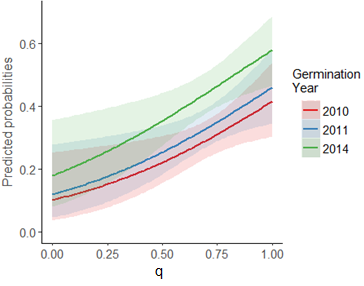


Figure S3. Predicted probability curves for each germination year of cutting success along the scale of *q* with 95% confidence intervals.


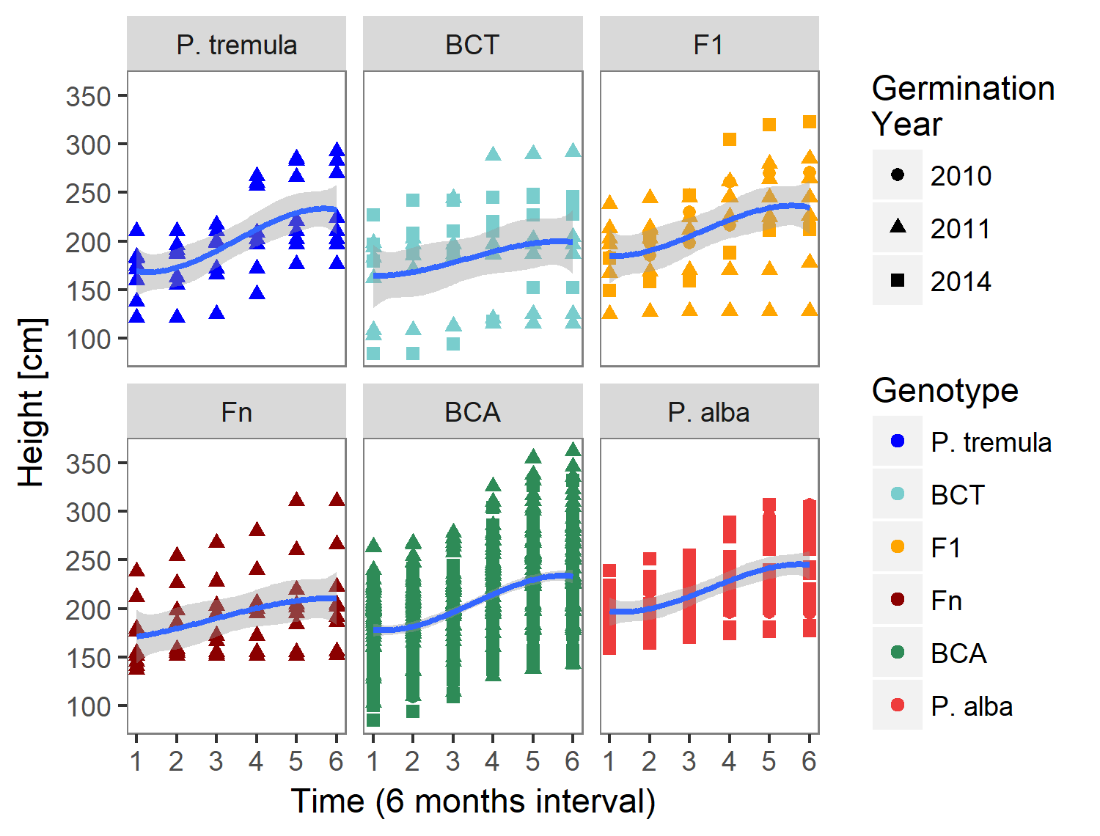


Figure S4. Polynomial regression line for the same model shown separately by genotype, representing changes in height during the six measurement time points.


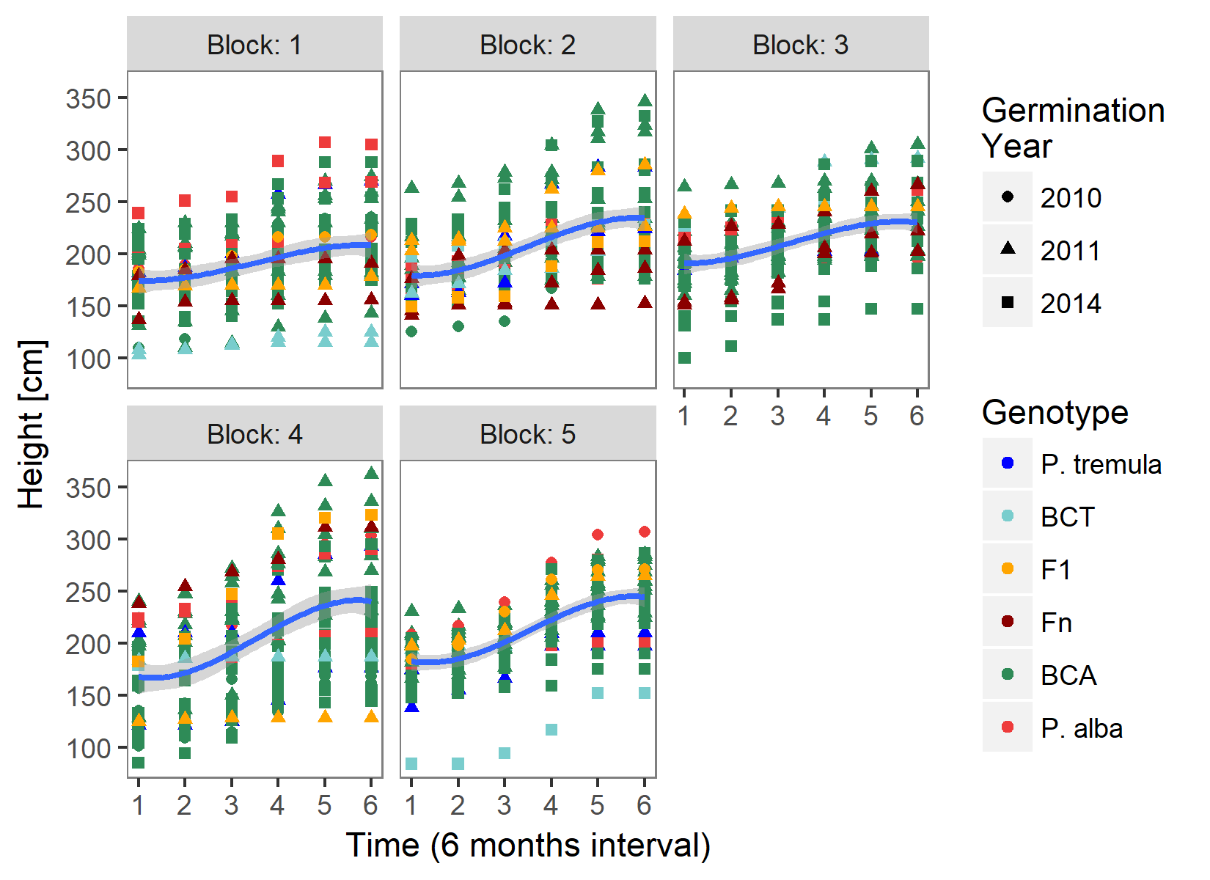


Figure S5. Scatterplot with 95% confidence interval of the best fitting model for height changes in each months, showing the effects of blocks on height.


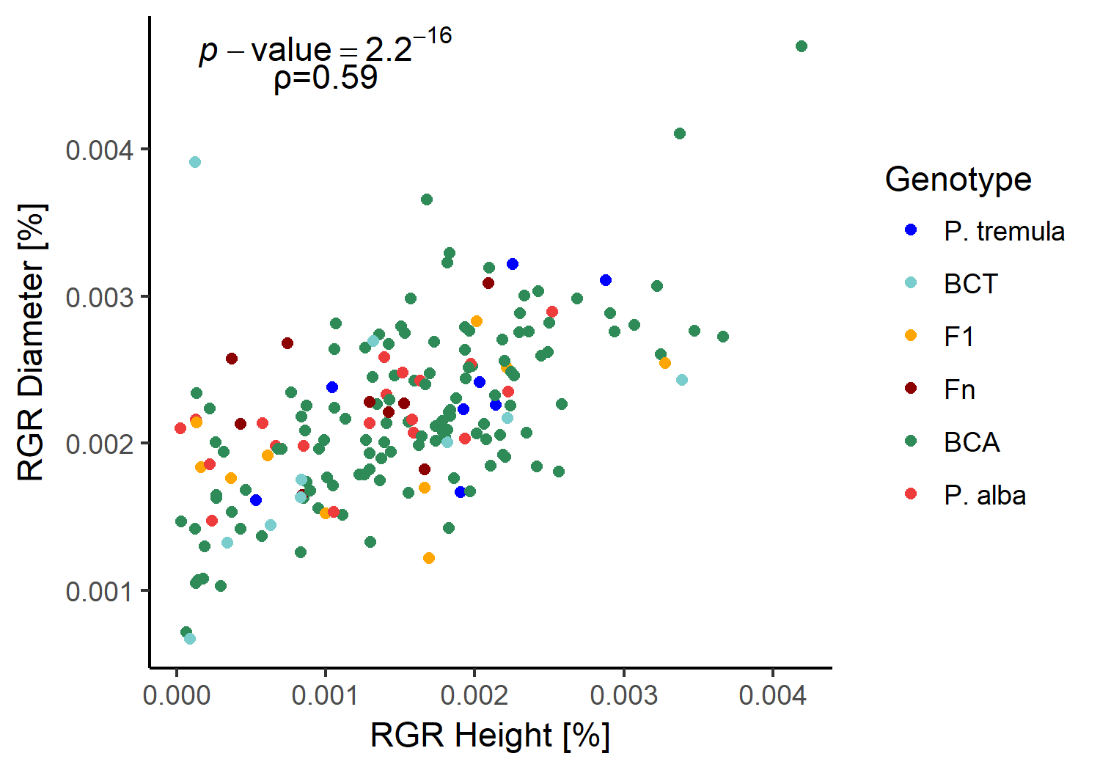


Figure S6. Plot of Spearman’s rank test shows a positive correlation between RGR calculated from height data and RGR from diameter.


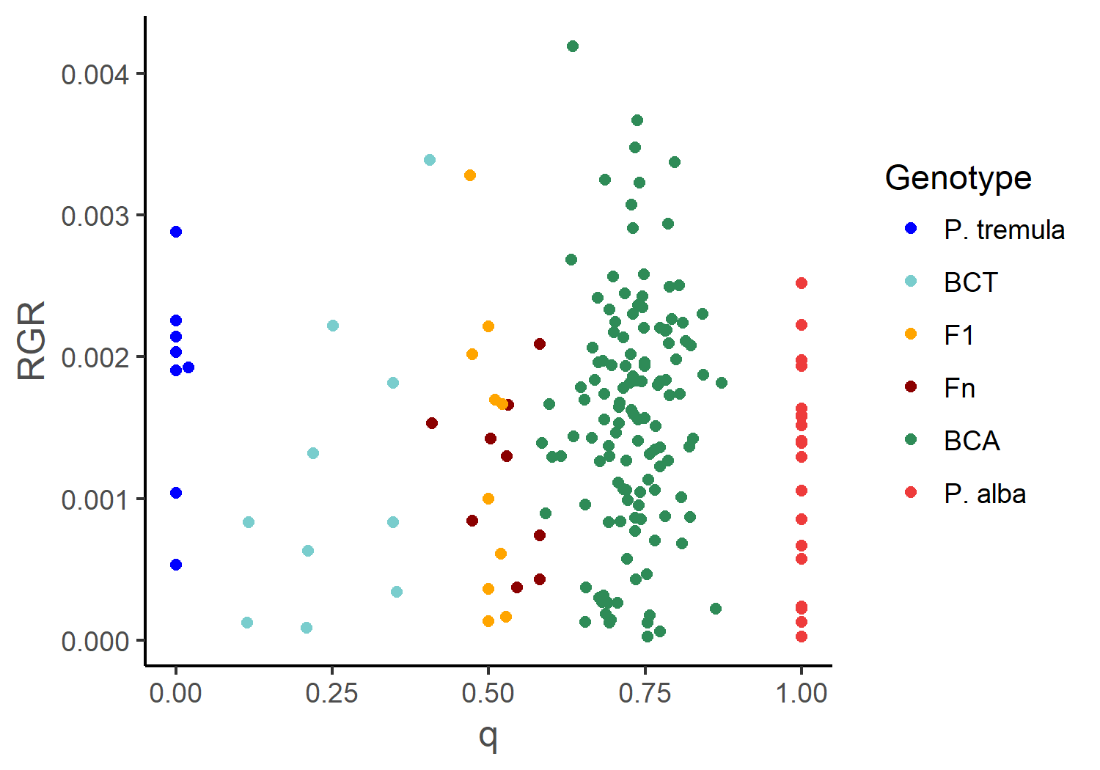


Figure S7. Relationship between *q* and overall RGR calculated for 2018.


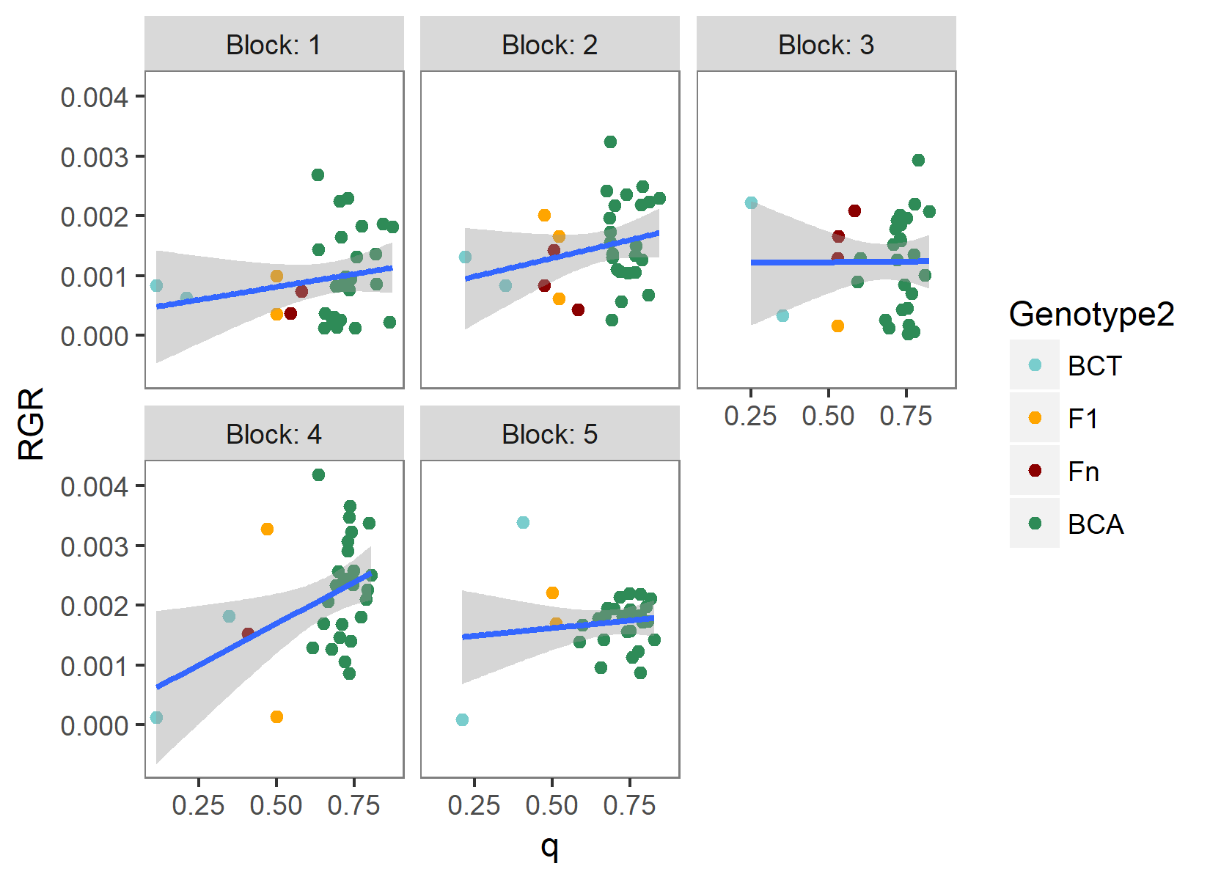


Figure S8. Regression line of the top rank model for overall RGR of hybrids showing effects of blocks in the CG.
